# Does acute malnutrition in young children increase the risk of treatment failure following artemisinin-based combination therapy? A WWARN individual patient data meta-analysis

**DOI:** 10.1016/S2214-109X(24)00003-2

**Published:** 2024-03-12

**Authors:** WWARN ACT Malaria and Malnutrition Study Group, Kasia Stepniewska, Richard Allan, Anupkumar R Anvikar, Thomas A Anyorigiya, Elizabeth A Ashley, Quique Bassat, Elisabeth Baudin, Anders Bjorkman, Maryline Bonnet, Caroline Boulton, Teun Bousema, Gwenaelle Carn, Verena I Carrara, Umberto D'Alessandro, Timothy ME Davis, Lise Denoeud-Ndam, Meghna Desai, Abdoulaye A Djimde, Grant Dorsey, Jean-François Etard, Catherine Falade, Caterina Fanello, Oumar Gaye, Raquel Gonzalez, Francesco Grandesso, Anastasia D Grivoyannis, Rebecca F Grais, Georgina S Humphreys, Deus S Ishengoma, Corine Karema, Kassoum Kayentao, Kalynn Kennon, PeterG Kremsner, Moses Laman, Ibrahim M Laminou, Eusebio Macete, Andreas Martensson, Mayfong Mayxay, Hervé IB Menan, Clara Menéndez, Brioni R Moore, Carolyn Nabasumba, Jean-Louis Ndiaye, Abel Nhama, Francois Nosten, Marie Onyamboko, Aung Pyae Phyo, Michael Ramharter, Philip J Rosenthal, Birgit Schramm, Yagya D Sharma, Sodiomon B Sirima, Nathalie Strub-Wourgaft, Khadime Sylla, Ambrose O Talisuna, Emmanuel A Temu, Julie I Thwing, Halidou Tinto, Giovanni Valentini, Nicholas J White, Adoke Yeka, Sheila Isanaka, Karen I Barnes, Philippe J Guerin

## Abstract

**Background:**

The geographical, demographic, and socioeconomic distributions of malaria and malnutrition largely overlap. It remains unknown whether malnutrition affects the efficacy of WHO-recommended artemisinin-based combination therapies (ACTs). A previous systematic review was inconclusive as data were sparse and heterogeneous, indicating that other methodological approaches, such as individual patient data meta-analysis, should be considered. The objective of this study was to conduct such a meta-analysis to assess the effect of malnutrition (wasting and stunting) on treatment outcomes in children younger than 5 years treated with an ACT for uncomplicated falciparum malaria.

**Methods:**

We conducted a meta-analysis of individual patient data from studies identified through a systematic review of literature published between 1980 and 2018 in PubMed, Global Health, and Cochrane Libraries (PROSPERO CRD42017056934) and inspection of the WorldWide Antimalarial Resistance Network (WWARN) repository for ACT efficacy studies, including children younger than 5 years with uncomplicated falciparum malaria. The association of either acute (wasting) or chronic (stunting) malnutrition with day 42 PCR-adjusted risk of recrudescence (ie, return of the same infection) or reinfection after therapy was investigated using Cox regression, and with day 2 parasite positivity using logistic regression.

**Findings:**

Data were included from all 36 studies targeted, 31 from Africa. Of 11 301 eligible children in 75 study sites, 11·5% were wasted (weight-for-height Z score [WHZ] <–2), and 31·8% were stunted (height-for-age Z score [HAZ] <–2). Decrease in WHZ was associated with increased risk of day 2 positivity (adjusted odds ratio 1·12, 95% CI 1·05–1·18 per unit; p=0·0002), treatment failure (adjusted hazard ratio [AHR] 1·14, 95% CI 1·02–1·26, p=0·016), and reinfection after therapy (AHR 1·09, 1·04–1·13, p=0·0003). Children with milder wasting (WHZ –2 to –1) also had a higher risk of recrudescence (AHR 1·85, 1·29–2·65, p=0·0008 *vs* WHZ ≥0). Stunting was not associated with reduced ACT efficacy.

**Interpretation:**

Children younger than 5 years with acute malnutrition and presenting with uncomplicated falciparum malaria were at higher risk of delayed parasite clearance, ACT treatment failure, and reinfections. Stunting was more prevalent, but not associated with changes in ACT efficacy. Acute malnutrition is known to impact medicine absorption and metabolism. Further study to inform dose optimisation of ACTs in wasted children is urgently needed.

**Funding:**

Bill & Melinda Gates Foundation.

**Translation:**

For the French translation of the abstract see Supplementary Materials section.

## Introduction

The geographical, demographic, and socioeconomic distributions of malaria and malnutrition largely overlap across tropical and subtropical regions of low-income and middle-income countries. Children younger than 5 years were estimated to account for 80% of all malaria deaths in 2021,^1^ and malnutrition accounts for nearly half of all deaths in children younger than 5 years.^2^, ^3^ After years of decline, global malaria cases and deaths have increased since 2015, as a result of various factors, including lower investments in malaria control programmes, antimalarial drug and insecticide resistance, and (more recently) the COVID-19 pandemic-related disturbance of health systems.^1^ The pandemic has also increased the burden of malnutrition, with dire predictions of a growing global food crisis precipitated by the war in Ukraine.^4^

Acute malnutrition can lead to sudden weight loss or failure to gain weight, and it can manifest rapidly in regions affected by food insecurity or humanitarian emergencies. Before the COVID-19 pandemic, in 2019, 47 million children were estimated to have acute malnutrition (wasting, defined as a low weight-for-height Z score [WHZ]; panel), of whom 14·3 million were severely wasted.^5^, ^6^ Over the past 3 years, there has been an estimated 30% increase in the number of children with wasting, affecting an additional 13·6 million children.^4^ Across the 41 malaria-endemic countries in sub-Saharan Africa in 2020, wasting was present in a median 5·7% (range 0·6–22·7) of children younger than 5 years,^5^ with an estimated 2·5 million overall malaria cases in wasted children.^6^ Chronic malnutrition can lead to linear growth flattening (stunting); although the associated risk of death is lower than for wasting, stunting is highly prevalent in resource-poor settings, with one in three young children in sub-Saharan Africa estimated to be stunted.^7^

Panel Malnutrition (undernutrition*) definitions *Overweight and obesity are not considered in this report

Malnutrition is a multifactorial condition generally related to poor nutrient intake, absorption, or utilisation. It can lead to various clinical presentations depending on the underlying factors and duration of exposure. A number of anthropometric measurements exist for children aged between 6 months and 5 years defining various types of malnutrition:

• Wasting: Acute undernutrition leads to rapid weight loss or failure to gain weight, defined by low weight-for-height Z score (WHZ). WHZ <–2 is referred to as wasting, and WHZ <–3 as severe wasting. Mid-upper-arm circumference <11·5 cm is another simple and low-cost indicator to detect severe acute malnutrition.
• Stunting: Chronic undernutrition resulting from inadequate nutrition over a longer period leads to failure of linear growth, defined by a low height-for-age Z score (HAZ). HAZ <–2 is referred to as stunting and HAZ <–3 as severe stunting.
• Underweight: A combination measure defined by a low weight-for-age Z score (WAZ), which occurs as a result of wasting or stunting. WAZ <–2 is referred to as underweight and WAZ <–3 as severe underweight.

**Research in context**

**Evidence before this study**

We previously conducted a systematic literature review of studies published between Jan 1, 1980, and Feb 19, 2018, to assess the association between malnutrition and the risk of malaria and the risk of antimalarial treatment failure. Database searches included PubMed, Global Health, and Cochrane Libraries, and articles published in English, French, or Spanish were reviewed. Search terms included those related to falciparum malaria, malnutrition, and anthropometric measures. 33 studies were eligible for inclusion; however, a meaningful aggregated data meta-analysis was not possible due to methodological heterogeneity of studies. Quality of the included studies was graded as a median of 5 (range 1–9) using the 9-point Newcastle-Ottawa Scale. Only five studies explored the effect of malnutrition on antimalarial treatment response, with contradictory results, and data on acute malnutrition (wasting) were scarce as height was seldom recorded. Because of several limitations (evaluated by the GRADE approach), including serious imprecision, and serious inconsistency of the evidence for all three forms of malnutrition (wasting, stunting, and underweight), this literature review was inconclusive to assess the association between malnutrition and malaria.

**Added value of this study**

In contrast to previous inconsistent findings on the effects of malnutrition on malaria treatment response, by combining available individual patient data from studies that included some malnourished children and measured both height and weight deposited in the WorldWide Antimalarial Resistance Network (WWARN) data platform, we demonstrated clearly the lower efficacy and post-treatment prophylaxis effect of artemisinin-based combination therapies (ACTs) in acutely malnourished children younger than 5 years. We did not find an association between stunting—which is the more prevalent form of malnutrition—with adverse ACT treatment response.

**Implications of all the available evidence**

Young children's height and weight or mid-upper arm circumference should be measured routinely in clinical practice and therapeutic antimalarial efficacy studies to identify children younger than 5 years who are at an increased risk of poorer malaria treatment response and thus require more careful follow-up. Optimisation of antimalarial treatment for acutely malnourished children is needed to decrease the negative impact of this comorbidity.

Current WHO guidelines for the treatment of malaria recommend the same weight-based artemisinin-based combination therapy (ACT) dosage regimens in malnourished and adequately nourished patients.^8^ However, pharmacokinetic parameters, such as bioavailability, might differ between malnourished and adequately nourished patients, and between those who are wasted and stunted,^9^, ^10^, ^11^ although data are scarce. Furthermore, malnourished children are excluded from the majority of antimalarial efficacy trials, including therapeutic efficacy studies following WHO protocols.^12^ A better understanding of the complex interaction between malnutrition and antimalarial efficacy is needed to inform risks of treatment failure and develop strategies to address malaria dosing regimens in malnourished children.

We previously conducted a systematic review of studies published between 1980 and 2018, to assess the evidence of interactions between malaria and malnutrition, namely the association between undernutrition and (1) the risk of malaria and (2) antimalarial treatment efficacy; however, the scant available evidence was inconsistent.^13^ The review highlighted that chronic undernutrition was frequently, but not consistently, associated with severity of malaria, such as high-density parasitaemia and anaemia. Although 33 studies were included in that review, only five explored the effect of malnutrition on antimalarial treatment response, with contradictory results.^9^, ^14^, ^15^, ^16^, ^17^ Data on wasting were scarce because height was seldom recorded.

The objective of this study was to conduct an individual patient data (IPD) meta-analysis to assess the effect of malnutrition (wasting and stunting) on treatment outcomes in children younger than 5 years treated with an ACT for uncomplicated falciparum malaria, using all available data from antimalarial efficacy studies that included young children and measured both their weight and height.

## Methods

### Search strategy and selection criteria

Since our previous systematic review of published literature (PROSPERO CRD42017056934) identified only five studies exploring malnutrition and antimalarial treatment outcomes and did not provide a sufficiently comprehensive dataset,^13^ this IPD meta-analysis also included relevant studies identified in the WorldWide Antimalarial Resistance Network (WWARN) repository.^18^ The WWARN repository contains standardised individual patient records from 320 antimalarial efficacy studies for *Plasmodium falciparum* malaria, enrolling patients from study sites in 69 countries with a diverse range of transmission intensities.

We searched for eligible studies among all antimalarial efficacy studies of uncomplicated *P falciparum* malaria that followed up patients prospectively for a minimum of 28 days; assessed efficacy of artemether–lumefantrine, artesunate–amodiaquine, dihydroartemisinin–piperaquine, or artesunate–mefloquine; included children younger than 5 years; and reported data on patient age, ACT dose, weight, sex, and height at inclusion, treatment outcome assessed by PCR genotyping, and study location. Data for these four, most widely used ACTs were previously systematically identified.^19^, ^20^, ^21^, ^22^ Permission for reuse of these data was obtained from the data contributors in accordance with the Infectious Diseases Data Observatory (IDDO) data access policy.^18^ Analyses included all available data, last extracted on Sept 28, 2020. The primary endpoint was the PCR-adjusted risk of *P falciparum* recrudescence (ie, return of the same infection) by day 42. Secondary endpoints consisted of the PCR-adjusted risk of *P falciparum* reinfection by day 42, and parasite positivity on day 2. See appendix 2 for definition of endpoints (appendix 2 pp 2–3) and for PCR genotyping methods used and treatment outcomes observed in each study (appendix 2 pp 5–7).

### Data analysis

Statistical analyses were carried out using Stata (version 17.1) according to an a-priori statistical analysis plan.^23^ A one-stage random effects IPD meta-analysis was conducted for each outcome, using Cox regression, with shared frailty^24^ to account for study-site, for PCR-adjusted risk of *P falciparum* recrudescence or *P falciparum* reinfection, and logistic regression with random study-site intercept, for parasite positivity on day 2. The exposures of primary interest, anthropometric measures (WHZ and height-for-age Z score [HAZ] at inclusion), were assessed using WHO standards^25^ and analysed separately. WHZ was evaluated using data from all eligible studies, while HAZ was assessed only in studies that measured age with adequate precision (ie, in months). Anthropometric measures were modelled as continuous, binary (eg, wasting defined as WHZ <–2), and categorical (eg, five categories defined by Z score thresholds of –3, –2, –1, and 0) variables. The effect of the following baseline covariates as potential confounders were examined: sex, log_10_ asexual parasite density, hyperparasitaemia (asexual parasitaemia >100 000 parasites per μL), haemoglobin, anaemia (severe: haemoglobin <7 g/dL; moderate: haemoglobin ≥7 g/dL but <10 g/dL), presence of or history of fever (temperature >37·5°C), geographical region, and malaria transmission intensity (low, moderate, or high). All models were adjusted for ACT treatment and age category (<1, 1, 2, 3, or 4 years old) to facilitate comparison of children with different nutritional status by age category. Differences in association between outcomes and anthropometric measures between children treated with different ACTs were explored using interaction terms. Models were not adjusted for mg/kg dose; instead, variables derived from mg/kg dose to define underdosing (yes or no) of the artemisinin derivative and partner drug were examined. Underdosing was defined as the total mg/kg actual dose (or per protocol dose if unavailable) below the WHO-recommended therapeutic range.^8^ The proportional hazard assumption in the Cox model was tested using Schoenfeld residuals. Wasting (recrudescence and reinfection models) and ACT treatment (reinfection model) did not satisfy assumption and separate estimates were obtained for time intervals 0–28 and 28–42 days, or 0–21, 21–28, and 28–42 days, as appropriate.

Bias related to individual studies was assessed using the modified Cochrane tool version 2^26^ for randomised controlled trials and the Newcastle-Ottawa Scale^27^ for non-randomised studies. In the sensitivity analysis, the final models were refitted (1) with each study's data excluded, one at a time, and a coefficient of variation around the parameter estimates calculated; (2) in studies that used at least three molecular markers to distinguish recrudescence from the reinfection, and (3) on imputed datasets using interval regression imputation methods for age in studies that did not specify age in months. For further details on covariates, model development, multiple imputations, and risk of bias assessment see appendix 2 (pp 2–4).

### Ethical approval

All data included in this analysis were obtained in accordance with the laws and ethical approvals applicable to the countries in which the studies were conducted and were obtained with the knowledge and consent of the patient's parent or guardian. Data were fully anonymised when shared with WWARN. The use of existing data that are fully anonymised and that researchers cannot trace back to identifiable individuals does not require the review of the Ethics Committee under the guidelines of the Oxford Central University Research Ethics Committee.

### Role of the funding source

The funder of the study had no role in study design, data collection, data analysis, data interpretation, or writing of the report.

## Results

Investigators of all 36 clinical trials identified for inclusion agreed to contribute their study meta-data and individual patient records. After exclusion of patients without *P falciparum* parasitaemia on enrolment, without follow-up data, or with missing age, weight, height, or sex information, a total of 11 301 patients contributed to the analysis dataset (figure; full list of studies in appendix 2 p 5). Data were collected between 2002 and 2015 from 75 study sites, with children followed up after initiation of treatment for 28 days in 22 of 36 studies, for 42 days in 12 studies, and for 63 days in two studies. (appendix 2 p 5). Malnourished children were not eligible for enrolment in 21 studies that used different definitions and severity of malnutrition for exclusion (appendix 2 p 6). Nevertheless, all studies enrolled some children with wasting. Distribution of children's age, weight, and height in individual studies are shown in appendix 2 (pp 6–7, 18). Forest plots of the unadjusted association between the anthropometric measures and treatment outcomes within studies are shown in appendix 2 (pp 19–20).

**Figure:**
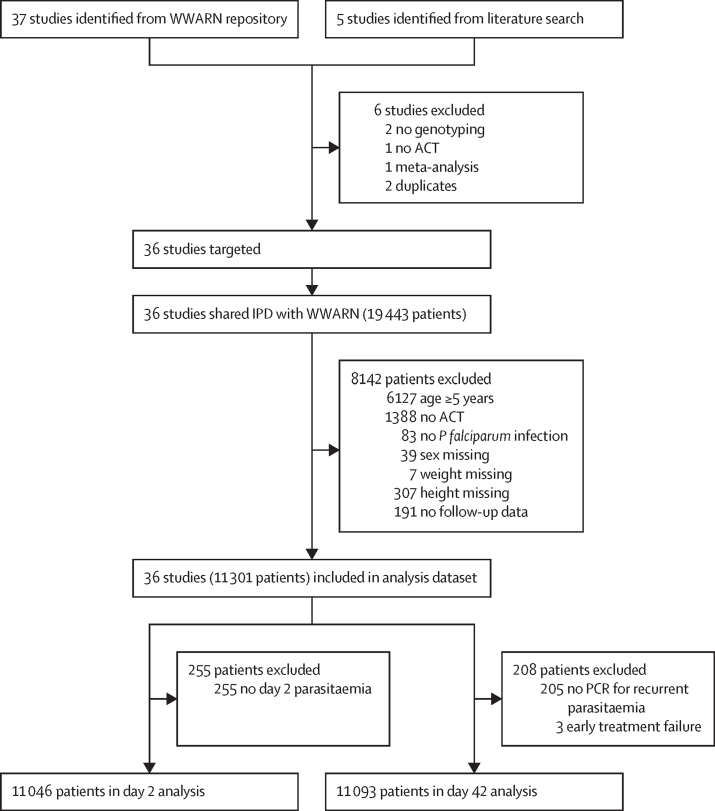
Study profile ACT=artemisinin-based combination therapy. IPD=individual patient data. WWARN=WorldWide Antimalarial Resistance Network.

Nearly all children (11 169 [98·8%] of 11 301) were recruited in sub-Saharan Africa, with the majority from eastern Africa (6128 [54·2%] of 11 301; table 1; appendix 2 p 21). Overall, 1284 (11·5%) of 11 189 children met the definition of wasting (WHZ <–2), and almost a third were stunted (HAZ <–2) (appendix 2 p 21). Children with wasting had similar baseline characteristics to children without wasting, except for lower haemoglobin levels and an increased prevalence of anaemia (table 1). Artemether–lumefantrine was the most commonly used ACT (5015 [44·4%] of 11 301 children; appendix 2 p 8); it was administered with food in 11 studies and without food in eight studies; food intake was unknown for eight studies. Underdosing was frequent for dihydroartemisinin–piperaquine (56·5%) and artemether–lumefantrine (30·6%), but was less than 5% for other ACTs (appendix 2 p 8). Children with wasting received significantly higher mg/kg doses of both the artemisinin derivative and partner drug than non-wasted children (all p<0·0030, adjusted for study site) for all ACTs except dihydroartemisinin–piperaquine and artesunate–amodiaquine fixed-dose combination (FDC; appendix 2 p 22).

**Table 1:** Baseline characteristics for patients in the analysis dataset

		**All children**		**Children with wasting**		**Children without wasting**	
		N	Median (range) or n (%)	N	Median (range) or n (%)	N	Median (range) or n (%)
Age, years		11 301	2·5 (0·2–4·9)	1284	2·1 (0·4–4·9)	9905	2·4 (0·2–4·9)
Age group							
	<1 year	11 301	1164 (10·3%)	1284	143 (11·1%)	9905	1014 (10·2%)
	1 year	11 301	3001 (26·6%)	1284	401 (31·2%)	9905	2577 (26·0%)
	2 years	11 301	2830 (25·0%)	1284	299 (23·3%)	9905	2513 (25·4%)
	3 years	11 301	2359 (20·9%)	1284	229 (17·8%)	9905	2097 (21·2%)
	4 years	11 301	1947 (17·2%)	1284	212 (16·5%)	9905	1704 (17·2%)
Sex							
	Male	11 301	5866 (51·9%)	1284	699 (54·4%)	9905	5102 (51·5%)
	Female	11 301	5435 (48·1%)	1284	585 (45·6%)	9905	4803 (48·5%)
Weight, kg		11 301	11 (5–28)	1284	9 (5–18)	9905	11·5 (5–27)
Height, cm		11 301	85 (37–140)	1284	85 (61–120)	9905	85 (51–120)
Haemoglobin, g/dL		11 107	9·3 (3·4–19·1)	1264	8·8 (5–13·8)	9732	9·3 (3·4–19·1)
Anaemia*							
	Severe	11 107	877 (7·9%)	1264	131 (10·4%)	9732	738 (7·6%)
	Moderate	11 107	7226 (65·1%)	1264	928 (73·4%)	9732	6232 (64·0%)
Temperature, °C		11 185	38 (34·5–41·2)	1279	38 (35·3–41·2)	9794	38 (34·5–41·1)
Fever†		11 294	7194 (63·7%)	1281	871 (68·0%)	9901	6250 (63·1%)
Log_10_ parasitaemia‡		11 301	4·4 (1·2–6·1)	1284	4·4 (1·5–5·5)	9905	4·5 (1·2–6·1)
Hyperparasitaemia§		11 301	1614 (14·3%)	1284	189 (14·7%)	9905	1411 (14·2%)
Presence of gametocytes		8867	987 (11·1%)	961	111 (11·6%)	7846	870 (11·1%)
Underweight¶							
	WAZ <–1	11 295	5631 (49·9%)	1284	1180 (91·9%)	9903	4419 (44·6%)
	WAZ <–2	11 295	2185 (19·3%)	1284	914 (71·2%)	9903	1249 (12·6%)
	WAZ <–3	11 295	578 (5·1%)	1284	401 (31·2%)	9903	165 (1·7%)
Wasting¶							
	WHZ <–1	11 189	3493 (31·2%)	0	NA	9905	2209 (22·3%)
	WHZ <–2	11 189	1284 (11·5%)	1284	1284 (100%)	0	NA
	WHZ <–3	11 189	381 (3·4%)	1284	381 (29·7%)	0	NA
Stunting¶							
	HAZ <–1	11 171	6761 (60·5%)	1279	721 (56·4%)	9838	6013 (61·1%)
	HAZ <–2	11 171	3557 (31·8%)	1279	390 (30·5%)	9838	3148 (32·0%)
	HAZ <–3	11 171	1322 (11·8%)	1279	105 (8·2%)	9838	1201 (12·2%)
MUAC, mm		1783	146 (85–190)	310	130 (95–190)	1456	150 (85–190)
Region							
	Central Africa	11 301	1336 (11·8%)	1284	14 (1·1%)	9905	50 (0·5%)
	Eastern Africa	11 301	6128 (54·2%)	1284	94 (7·3%)	9905	1228 (12·4%)
	Western Africa	11 301	3705 (32·8%)	1284	446 (34·7%)	9905	5616 (56·7%)
	Asia	11 301	65 (0·6%)	1284	9 (0·7%)	9905	57 (0·6%)
	Melanesia	11 301	67 (0·6%)	1284	721 (56·2%)	9905	2954 (29·8%)
Transmission intensity area‖							
	High	11 301	4379 (38·7%)	1284	623 (48·5%)	9905	3737 (37·7%)
	Moderate	11 301	4460 (39·5%)	1284	356 (27·7%)	9905	4033 (40·7%)
	Low	11 301	2462 (21·8%)	1284	305 (23·8%)	9905	2135 (21·6%)

Numbers of children who are wasted and not wasted do not add up to the total number of children, as WHZ is out of range for 112 children. Wasting defined as WHZ <–2. HAZ=height-for-age Z score. MUAC=mid-upper arm circumference. N=number of evaluated children. n=number with outcome. NA=not applicable. WAZ=weight-for-age Z score. WHZ=weight-for-height Z score.

* Severe anaemia defined as haemoglobin <7 g/dL, moderate anaemia as haemoglobin ≥7 g/dL but <10 g/dL.

† Defined as temperature >37·5°C or history of fever.

‡ Parasitaemia is measured as parasites per μL.

§ Defined as parasitaemia >10^5^/μL.

¶ Groups overlap, groups defined by lower cutoff are included in the group defined by the higher cutoff.

‖ Malaria transmission intensity defined from estimates of *Plasmodium falciparum* prevalence rate (PfPR) according to enrolment year and location, assuming low transmission for study sites with a PfPR <0·10, moderate transmission if PfPR 0·10 to <0·40, and high transmission if PfPR ≥0·40.

Parasitaemia remained detectable in 1029 (9·2%) of 11 233 children on day 2 and 96 (0·9%) of 11 270 children on day 3, with similar percentages between wasted and not-wasted children. However, all children with a WHZ less than 0 had an increased risk of parasite positivity on day 2 with decreasing WHZ, when compared with those with a higher WHZ (adjusted odds ratio [AOR] 1·12, 95% CI 1·05–1·18, p=0·0002 per unit decrease; table 2, table 3, appendix 2 pp 9–10), partially explaining why the risk was similar in children with and without wasting (when defined as a WHZ <–2). Compared with children treated with artemether–lumefantrine, parasite positivity after treatment was lower among those treated with dihydroartemisinin–piperaquine (AOR 0·79, 95% CI 0·63–0·98, p=0·030) or artesunate–amodiaquine FDC (AOR 0·47, 0·37–0·60, p<0·0001). However, the effect of wasting on early parasitological response was similar between ACTs. In the multivariable model, neither age nor artemisinin derivative underdosing were associated with day 2 parasitaemia, but severe anaemia was associated with increased risk of day 2 parasitaemia (table 2).

**Table 2:** Independent predictors of treatment outcomes during 42 days of follow-up, from final multivariable regression models

		**Parasite positivity on day 2**			**Recrudescence**			**Reinfection**		
		N (n)	AOR (95% CI)	p value	N (n)	AHR (95% CI)	p value	N (n)	AHR (95% CI)	p value
Wasting—WHZ (decrease)*		10 747 (983)	1·12 (1·05–1·18)	0·0002	10 775 (305)	1·14 (1·02–1·26)†	0·016	10 789 (1864)	1·09 (1·04–1·13)‡	0·0003
ACT§										
	Artemether–lumefantrine	4606 (455)	1	NA	4725 (142)	1	NA	4724 (966)	1	NA
	Artesunate–amodiaquine (FDC)	2248 (172)	0·47 (0·37–0·60)	<0·0001	2222 (41)	0·68 (0·47–1·01)	0·054	2222 (411)	0·86 (0·75–0·98)	0·029
	Artesunate–amodiaquine (non-FDC)	572 (55)	1·17 (0·62–2·21)	0·62	564 (30)	2·13 (1·25–3·61)	0·0052	564 (129)	2·20 (1·50–3·23)	0·0001
	Artesunate–mefloquine	518 (30)	0·80 (0·48–1·34)	0·40	485 (11)	0·79 (0·39–1·60)	0·52	499 (129)	0·84 (0·66–1·06)	0·14
	Dihydroartemisinin–piperaquine	2802 (271)	0·79 (0·63–0·98)	0·030	2779 (81)	0·61 (0·45–0·84)	0·0022	2780 (229)	0·36 (0·30–0·42)	<0·0001
Age group										
	<1 year	1114 (83)	0·90 (0·65–1·24)	0·51	1136 (26)	1·14 (0·67–1·93)	0·63	1136 (161)	0·64 (0·52–0·78)	<0·0001
	1 year	2847 (240)	1·05 (0·83–1·34)	0·69	2892 (90)	1·46 (0·97–2·18)	0·067	2893 (537)	0·84 (0·72–0·98)	0·024
	2 years	2691 (274)	1·18 (0·94–1·49)	0·15	2681 (93)	1·59 (1·07–2·36)	0·021	2684 (508)	0·99 (0·85–1·15)	0·88
	3 years	2247 (214)	1·00 (0·78–1·26)	0·98	2238 (60)	1·29 (0·85–1·95)	0·24	2242 (370)	0·96 (0·82–1·12)	0·63
	4 years	1847 (172)	1	NA	1828 (36)	1	NA	1834 (288)	1	NA
Sex										
	Male	NS	NS	NS	NS	NS	NS	6500 (1010)	1·10 (1·01–1·21)	0·035
	Female	NS	NS	NS	NS	NS	NS	5189 (854)	1	NA
Transmission intensity area										
	High	4068 (276)	1·29 (0·72–2·31)	0·39	4194 (129)	1·15 (0·69–1·91)	0·59	3194 (1091)	2·08 (1·32–3·27)	0·0016
	Moderate	2247 (330)	0·59 (0·38–0·93)	0·023	2223 (76)	1·05 (0·68–1·64)	0·82	2224 (314)	1·14 (0·84–1·56)	0·40
	Low	4431 (377)	1	NA	4358 (100)	1	NA	4371 (314)	1	NA
Log_10_ parasitaemia¶		10 746 (983)	2·31 (1·98–2·71)	<0·0001	10 775 (305)	1·32 (1·06–1·64)	0·012	NA	NA	NA
Anaemia‖										
	No	3779 (345)	1	NA	3759 (88)	1	NA	3768 (564)	1	NA
	Moderate	6131 (544)	1·03 (0·87–1·22)	0·72	6167 (178)	1·15 (0·88–1·51)	0·30	6171 (1114)	1·08 (0·97–1·21)	0·15
	Severe	836 (94)	1·52 (1·13–2·03)	0·0054	849 (39)	1·67 (1·12–2·49)	0·012	850 (186)	1·25 (1·05–1·49)	0·012
Fever**										
	No	3983 (252)	1	NA	NS	NS	NS	NS	NS	NS
	Yes	6763 (731)	1·37 (1·15–1·63)	0·0005	NS	NS	NS	NS	NS	NS
Partner drug underdose										
	No	NS	NS	NS	7731 (193)	1	NA	NS	NS	NS
	Yes	NS	NS	NS	3044 (112)	1·55 (1·16–2·06)	0·0028	NS	NS	NS

The table shows 28-day follow-up estimates for wasting; estimates for 28–42 days are shown in footnotes. ACT=artemisinin-based combination therapy. AHR=adjusted hazard ratio. AOR=adjusted odds ratio. FDC=fixed-dose combination. N=number of evaluated children. n=number with outcome. NA=not applicable. NS=not selected (not selected in the final model as this variable did not improve the model significantly). WHZ=weight-for-height Z score.

* Estimates shown for 1 unit decrease in WHZ.

† AHR shown is for the first 28 days of follow-up; AHR 0·93 (0·78–1·10), p=0·32 was estimated for the interval of 28–42 days.

‡ AHR shown is for the first 28 days of follow-up; AHR 0·94 (0·88–1·01), p=0·071 was estimated for the interval of 28–42 days.

§ AHR is not constant across the follow-up time (for details see appendix 2 pp 13–14).

¶ Parasitaemia is measured as parasites per μL.

‖ Severe anaemia defined as haemoglobin <7 g/dL, moderate anaemia as haemoglobin ≥7 g/dL but <10 g/dL.

** Fever defined as temperature >37·5°C or history of fever.

**Table 3:** Association between malnutrition and treatment outcomes during 42 days follow-up, for different parameter representations

		**Parasite positivity on day 2**			Recrudescence*			Reinfection*		
		N (n)	AOR (95% CI)	p value	N (n)	AHR (95% CI)	p value	N (n)	AHR (95% CI)	p value
**Wasting**										
WHZ (decrease)†		10 747 (983)	1·12 (1·05–1·18)	0·0002	10 775 (305)	1·14 (1·02–1·26)	0·016	10 789 (1864)	1·09 (1·04–1·13)	0·0003
Wasted (WHZ <–2)										
	No	9580 (891)	1	NA	9546 (272)	1	NA	9553 (1587)	1	NA
	Yes	1166 (92)	1·16 (0·90–1·51)	0·256	1229 (33)	1·15 (0·77–1·74)	0·50	1236 (277)	1·32 (1·13–1·54)	0·0003
WHZ										
	≥0	4096 (398)	1	NA	4053 (95)	1	NA	4056 (578)	1	NA
	≥–1 to 0	3372 (316)	1·32 (1·10–1·58)	0·0027	3367 (97)	1·27 (0·90–1·79)	0·18	3370 (609)	1·05 (0·91–1·21)	0·51
	≥–2 to −1	2112 (177)	1·33 (1·08–1·65)	0·0088	2126 (80)	1·85 (1·29–2·65)	0·0008	2127 (400)	1·14 (0·98–1·34)	0·09
	≥–3 to −2	853 (64)	1·32 (0·96–1·82)	0·088	859 (27)	1·68 (1·02–2·75)	0·041	865 (202)	1·42 (1·17–1·72)	0·0004
	<–3	313 (28)	1·52 (0·96–2·42)	0·074	370 (6)	1·08 (0·43–2·74)	0·86	371 (75)	1·33 (0·99–1·79)	0·055
**Stunting**‡										
HAZ (decrease)§		9726 (879)	0·96 (0·91–1·01)	0·14	9777 (283)	0·99 (0·91–1·07)	0·74	9777 (1833)	0·99 (0·95–1·02)	0·44
Stunted (HAZ <–2)										
	No	6511 (580)	1	NA	6546 (191)	1	NA	6547 (1171)	1	NA
	Yes	3215 (299)	0·95 (0·79–1·13)	0·54	3231 (92)	0·90 (0·70–1·16)	0·43	3230 (580)	0·99 (0·90–1·10)	0·87
HAZ										
	≥0	1439 (137)	1	NA	1439 (34)	1	NA	1439 (253)	1	NA
	≥–1 to 0	2205 (186)	0·97 (0·74–1·26)	0·79	2208 (62)	1·11 (0·73–1·69)	0·64	2209 (407)	0·96 (0·82–1·13)	0·65
	≥–2 to −1	2867 (257)	0·96 (0·74–1·24)	0·74	2899 (95)	1·26 (0·85–1·88)	0·26	2899 (511)	0·87 (0·74–1·01)	0·072
	≥–3 to −2	2011 (176)	0·97 (0·74–1·29)	0·86	2041 (59)	1·07 (0·70–1·65)	0·75	2040 (379)	0·92 (0·78–1·08)	0·29
	<–3	1204 (123)	0·83 (0·61–1·13)	0·24	1190 (33)	1·00 (0·61–1·64)	0·99	1190 (201)	0·92 (0·76–1·11)	0·38

All models were adjusted for other independent predictors, as shown in table 2. AOR=adjusted odds ratio. AHR=adjusted hazard ratio. HAZ=height-for-age Z score. N=number of evaluated children. n=number with outcome. NA=not applicable. WHZ=weight-for-height Z score.

* For WHZ, AHR estimate for 28 days follow-up is shown; no significant association was observed for 28–42-day intervals, as presented in table 2.

† Estimates shown for 1 unit decrease in WHZ.

‡ 20 studies with age measured in months were included in the analysis.

§ Estimates shown for 1 unit decrease in HAZ.

Among 11 189 children with a WHZ, 325 (2·9%) had PCR-confirmed recrudescence (11 after day 42). The risk of recrudescence increased with wasting (table 2, table 3) in the first 28 days after treatment (adjusted hazard ratio [AHR] 1·14, 95% CI 1·02–1·26 per unit decrease in WHZ, p=0·016). This corresponds to AHRs of 1·58 (1·04–2·38), 1·41 (1·03–1·92), and 1·26 (1·02–1·54), respectively, when comparing a similar child with a WHZ of –4, –3, or –2 with a child with a score of 0 or higher. Children with a WHZ between –2 and –1 also had an increased risk of recrudescence by day 28 (AHR 1·85, 95% CI 1·29–2·65; table 3) compared with children with WHZ of 0 or higher, again partially explaining why the risk was similar in children with and without wasting (when defined as a WHZ <–2). No difference between ACTs in the effect of wasting on treatment efficacy was observed. Other variables associated with the increased risk of recrudescence during 42-day follow-up were high baseline parasitaemia, ACT partner drug underdosing, and severe anaemia at inclusion (table 2, appendix 2 pp 10–11). The risk of recrudescence initially increased with age, reaching the highest level for those aged 2 years (AHR 1·59, 95% CI 1·07–2·36 compared with those aged 4 years).

Among 11 189 children with a WHZ, 1947 (17·4%) had PCR-confirmed *P falciparum* reinfection (67 after day 42). Children with wasting had a higher risk of reinfection during 28-day follow-up (AHR 1·32, 95% CI 1·13–1·54, p=0·0003) compared with children without wasting (table 3). Lower WHZ was associated with an increased risk of reinfection during 28-day follow-up (AHR 1·09, 95% CI 1·04–1·13 per unit decrease, p=0·0003; table 2, appendix 2 pp 12–13). The effect of WHZ was similar between different ACTs. However, compared with artemether–lumefantrine, artesunate–amodiaquine FDC (AHR 0·86, 95% CI 0·75–0·98, p=0·029) and dihydroartemisinin–piperaquine (AHR 0·36, 0·30–0·42, p<0·0001) were associated with a lower risk of reinfection, and artesunate–amodiaquine non-FDC (AHR 2·20 (1·50–3·23), p=0·0001) with a higher risk of reinfection. This effect size varied over time, partly explained by different partner drug elimination half-lives (appendix 2 pp 13–14). Children presenting with severe anaemia were more likely to be reinfected (AHR 1·25, 1·05–1·49, p=0·012) compared with non-anaemic children. The lowest risk of reinfection was observed in infants, with risk increasing until about 2 years of age (table 2).

We did not observe any association between HAZ or different severity levels of stunting and treatment outcomes in univariable analysis (appendix 2 pp 9–13) or after adjusting for other covariates (table 3).

Most (24 of 36) studies were randomised, using computer-generated randomisation lists and concealed patient allocation; the remainder were single-arm studies (appendix 2 pp 15–16). In 29 studies, more than 85% of children enrolled could be included in this meta-analysis, since few age, sex, weight, or height variables were missing. In most studies, nearly all patients completed their follow-up; however, one study reported more than 10% of children being lost to follow-up before the end of the study. Two studies reported more than 10% of children having missing parasitaemia data on day 2. WHZ, a primary exposure, was evaluable in more than 90% of children for all but one study, where 28% of children were without a valid WHZ. Different genotyping methods to distinguish recrudescence and reinfection were used across studies. Estimates of effect sizes for the WHZ and HAZ remained very similar when the final models were refitted in the 21 studies with the most detailed molecular analysis (appendix 2 p 25).

Exclusion of one study at a time and refitting the multivariable models led to similar estimates of the effect of WHZ on day 2 parasite positivity (coefficient of variation 5·6%), recrudescence and reinfection after 28-day follow-up (coefficient of variation 11·2% and 4·7%, respectively), and of HAZ (appendix 2 pp 23–24). Analysis of age-imputed data showed similar results to the main analysis (appendix 2 p 26).

## Discussion

This IPD meta-analysis assessed the impact of acute and chronic malnutrition in more than 11 000 children younger than 5 years treated with an ACT for uncomplicated *P falciparum* malaria. Children with acute malnutrition (wasting) presenting with uncomplicated *P falciparum* malaria and treated with the WHO-recommended ACT dose had an increased risk of delayed parasite clearance, treatment failure (recrudescence), and new infection after therapy, compared with non-wasted children. Even mildly wasted children (WHZ –2 to 0), often excluded from standard definitions of wasting, were at increased risk of both delayed parasite clearance and treatment failure. The increased risk of treatment failure seen in those with a WHZ between –2 and –1 was nearly double that of children with a WHZ of 0 or higher (AHR 1·85; 95% CI 1·29–2·65; p=0·0008). We did not find any association of poorer ACT treatment responses with stunting, which is more prevalent than wasting.

One factor contributing to the adverse treatment responses observed among wasted young children could be their inadequate immune response. Children with wasting can have weakened immunity and are at a substantially increased risk of death from infectious diseases,^28^ including malaria. Severely wasted children with malaria have fewer classic clinical signs of the disease than non-malnourished children and often present with no fever or with hypothermia, delaying diagnosis and treatment.^29^ The increased risk of treatment failure in wasted young children might also be attributable to altered pharmacokinetic properties of the artemisinin derivative or the ACT partner drug.^10^, ^17^, ^30^ Reduced exposure to the artemisinin derivative might be associated with delayed parasite clearance (detectable parasitaemia on day 2), while the risk of treatment failure and duration of post-treatment prophylaxis (risk of or time to reinfection) depends primarily on the longer-acting partner drugs. However, there are scant data available for ACTs.

Previous smaller studies on the impact of nutritional status on malaria treatment outcomes among malnourished children have provided inconsistent results. In a cohort study of 351 children younger than 2 years in Uganda,^14^ 43% were stunted and 13% underweight. Among the 50 HIV-uninfected, unexposed children who were randomly assigned to treatment with dihydroartemisinin–piperaquine for uncomplicated malaria, a decreasing HAZ was associated with a higher risk of day 28 recurrent parasitaemia (HR 2·89, 95% CI 1·06–7·89 for HAZ ≥–2 to <–1 and HR 3·18, 1·18–8·56 for HAZ <–2, compared with children with HAZ >0). However, no evidence of reduced efficacy was observed among 445 children younger than 5 years in the Democratic Republic of the Congo,^15^ comparing the efficacy of standard doses of artesunate–amodiaquine between 61 children with and 369 without severe acute malnutrition (SAM); SAM was defined as a WHZ less than –3 (17 children) or the presence of nutritional oedema (56 children). A multicentre, open-label trial compared the efficacy of artemether–lumefantrine in 133 children with SAM versus 266 children without SAM in Mali and Niger; SAM was defined as a WHZ less than –3 or mid-upper arm circumference less than 115 mm, and day 42 recrudescence rate was similar between these two groups.^16^ However, the population pharmacokinetic evaluation showed that children with SAM were at higher risk of underexposure to lumefantrine and an increased risk of malaria reinfection (HR 2·10, 95% CI 1·04–4·22) than better-nourished children, despite only children with SAM receiving a high-fat nutritional supplement that should increase lumefantrine absorption substantially.^10^

Previous WWARN IPD meta-analyses showed associations between lower weight-for-age Z score (WAZ) and adverse artemether–lumefantrine treatment outcomes.^17^, ^19^ In one study, the risk of recrudescence was greatest in underweight African children aged 1–3 years compared with age-matched non-underweight children.^19^ In another IPD meta-analysis including 2787 patients treated with artemether–lumefantrine for uncomplicated falciparum malaria, the dose-adjusted day 7 lumefantrine concentrations were lowest in underweight children younger than 3 years, by 23% (95% CI −1 to 41) compared with adequately nourished children of the same age, and by 53% (37 to 65) compared with adults.^17^ The risk of reinfection increased with a decrease in WAZ among children aged 1–4 years in high-transmission areas, with an HR of 1·63 (95% CI 1·09–2·44) for a child with a WAZ of –3 compared with an adequately nourished child (WAZ=0). Neither of these studies was able to distinguish which of these underweight children were wasted or stunted, as data on height were not available.

In a study conducted in northern Ghana,^31^ the prophylactic effect of intermittent preventive treatment in infants (IPTi) with sulfadoxine–pyrimethamine in malnourished babies (who were either wasted, stunted, or underweight) was roughly half of that observed in non-malnourished infants. The relative contributions of inadequate bioavailability versus immune suppression could not be assessed, as sulfadoxine–pyrimethamine drug exposure was not measured in these infants. These findings are of particular concern given how widely sulfadoxine–pyrimethamine is used for malaria prevention in young children, both as IPTi and more widely administered with amodiaquine as seasonal (or perennial) malaria chemoprevention.^32^

The emergence and spread of resistance to artemisinin derivatives and partner drugs in Asia and the recent emergence of clinically significant artemisinin partial resistance in at least four African countries^1^, ^33^, ^34^ are reminders that the gains recorded over the last decades remain fragile. Before resistance to artemisinins and lumefantrine increase, alternative ACT dosing strategies need to be considered for wasted children to ensure favourable treatment outcomes and to limit spread of resistance.

This IPD meta-analysis had several limitations. The group of wasted children in this dataset might not be entirely representative of wasted young children. Since many studies excluded malnourished children by variable definitions, their population is under-represented, and our meta-analysis might include only less severe cases. Similarly, the severely malnourished children included might not have been typical, as they were not detected at screening. This could possibly explain why a linear trend for WHZ categories was not observed. No data were collected that measured immunity, and most studies did not measure drug exposure. Thus, it is not possible to disentangle these causal pathways, and the potential for dose optimisation to improve ACT treatment response in malnourished young children remains unknown. Lastly, almost half of the children included were treated with artemether–lumefantrine; the sample size for other ACTs was more limited. Further data collection is needed to understand the effects of wasting on outcomes with other ACTs and the relative contributions of inadequate bioavailability versus reduced immunity.

In contrast to previous inconsistent findings reported in the literature on the effects of malnutrition on malaria treatment response, by combining all available data from studies that included malnourished children and measured both height and weight, the lower treatment efficacy and post-treatment prophylaxis effects of ACTs in wasted children compared with non-wasted children younger than 5 years were clearly demonstrated by conducting an IPD meta-analysis. We did not find any association of stunting with adverse ACT treatment response. Most malaria therapeutic efficacy studies do not record children's height or mid-upper arm circumference, so identification of acute malnutrition is rarely done in therapeutic efficacy studies or clinical practice. Routinely capturing these data and calculating simple anthropometric measures would identify wasted children who are at an increased risk of poorer malaria treatment response, and thus need closer monitoring of adherence and treatment response. To improve future outcomes of acutely malnourished malaria-infected children, optimised ACT regimens need to be investigated.

## Data sharing

The data that support the findings of this study are available for access via the WorldWide Antimalarial Resistance Network (https://www.iddo.org/wwarn). Requests for access will be reviewed by a Data Access Committee to ensure that use of data protects the interests of the participants and researchers according to the terms of ethics approval and principles of equitable data sharing. Requests can be submitted by email to malariaDAC@iddo.org via the Data Access Form available at https://www.iddo.org/wwarn/accessing-data. The WWARN platform is registered with the Registry of Research Data Repositories (https://www.re3data.org/).

## Declaration of interests

We declare no competing interests.

## Supplementary Materials

French translation of the abstract

## References

[1] WHO (2021).

[2] Black RE, Victora CG, Walker SP (2013). Maternal and child undernutrition and overweight in low-income and middle-income countries. Lancet.

[3] UNICEF Child malnutrition. https://data.unicef.org/topic/nutrition/malnutrition/.

[4] Osendarp S, Verburg G, Bhutta Z (2022). Act now before Ukraine war plunges millions into malnutrition. Nature.

[5] UNICEF, WHO, World Bank Group (2021). Joint child malnutrition estimates—levels and trends—2021 edition. https://www.who.int/news/item/06-05-2021-the-unicef-who-wb-joint-child-malnutrition-estimates-group-released-new-data-for-2021.

[6] Takyi A, Carrara VI, Dahal P (2023). Characterisation of populations at risk of sub-optimal dosing of artemisinin-based combination therapy in Africa. PLoS Glob Public Health.

[7] Quamme SH, Iversen PO (2022). Prevalence of child stunting in sub-Saharan Africa and its risk factors. Clin Nutr Open Sci.

[8] WHO (2021).

[9] Obua C, Ntale M, Ogwal-Okeng JW, Gustafsson LL, Hellgren U, Petzold MG (2008). Impact of nutritional status on fixed-dose chloroquine and sulfadoxine/pyrimethamine combination treatment of malaria in Ugandan children. Int J Trop Med.

[10] Chotsiri P, Denoeud-Ndam L, Baudin E (2019). Severe acute malnutrition results in lower lumefantrine exposure in children treated with artemether-lumefantrine for uncomplicated malaria. Clin Pharmacol Ther.

[11] Krishnaswamy K (1989). Drug metabolism and pharmacokinetics in malnourished children. Clin Pharmacokinet.

[12] WHO (2009).

[13] Das D, Grais RF, Okiro EA (2018). Complex interactions between malaria and malnutrition: a systematic literature review. BMC Med.

[14] Verret WJ, Arinaitwe E, Wanzira H (2011). Effect of nutritional status on response to treatment with artemisinin-based combination therapy in young Ugandan children with malaria. Antimicrob Agents Chemother.

[15] Mitanlgala NP, D'Alessandro U, Hennart P, Donnen P, Porignon D (2012). Efficacy of artesunate plus amodiaquine for treatment of uncomplicated clinical falciparum malaria in severely malnourished children aged 6–59 months, Democratic Republic of Congo. J Clin Exp Pathol.

[16] Denoeud-Ndam L, Dicko A, Baudin E (2016). Efficacy of artemether-lumefantrine in relation to drug exposure in children with and without severe acute malnutrition: an open comparative intervention study in Mali and Niger. BMC Med.

[17] WorldWide Antimalarial Resistance Network (WWARN) Lumefantrine PK/PD Study Group (2015). Artemether-lumefantrine treatment of uncomplicated *Plasmodium falciparum* malaria: a systematic review and meta-analysis of day 7 lumefantrine concentrations and therapeutic response using individual patient data. BMC Med.

[18] WorldWide Antimalarial Resistance Network Accessing data. https://www.iddo.org/wwarn/accessing-data.

[19] Worldwide Antimalarial Resistance Network (WWARN) AL Dose Impact Study Group (2015). The effect of dose on the antimalarial efficacy of artemether–lumefantrine: a systematic review and pooled analysis of individual patient data. Lancet Infect Dis.

[20] WorldWide Antimalarial Resistance Network (WWARN) DP Study Group (2013). The effect of dosing regimens on the antimalarial efficacy of dihydroartemisinin-piperaquine: a pooled analysis of individual patient data. PLoS Med.

[21] Adjuik MA, Allan R, Anvikar AR (2015). The effect of dosing strategies on the therapeutic efficacy of artesunate-amodiaquine for uncomplicated malaria: a meta-analysis of individual patient data. BMC Med.

[22] Mansoor R, Dahal P, Humphreys GS, Guerin P, Ashley EA, Stepniewska K (2019). The effect of dose on the antimalarial efficacy of artesunate-mefloquine against *Plasmodium falciparum* malaria: a protocol for systematic review and individual patient data (IPD) meta-analysis. BMJ Open.

[23] WorldWide Antimalarial Resistance Network ACT Malaria and Malnutrition Study Group (2019). Statistical analysis plan. https://www.wwarn.org/tools-resources/act-malaria-and-malnutrition-study-group-statistical-analysis-plan.

[24] Higgins JP, Thompson SG, Spiegelhalter DJ (2009). A re-evaluation of random-effects meta-analysis. J R Stat Soc Ser A Stat Soc.

[25] de Onis M, Garza C, Victora CG, Onyango AW, Frongillo EA, Martines J (2004). The WHO Multicentre Growth Reference Study: planning, study design, and methodology. Food Nutr Bull.

[26] Sterne JAC, Savović J, Page MJ (2019). RoB 2: a revised tool for assessing risk of bias in randomised trials. BMJ.

[27] Wells GA, Shea B, O'Connell D The Newcastle-Ottawa Scale (NOS) for assessing the quality of non-randomised studies in meta-analyses. Ottawa, ON: Ottawa Hospital Research Institute. https://www.ohri.ca//programs/clinical_epidemiology/oxford.asp.

[28] Walson JL, Berkley JA (2018). The impact of malnutrition on childhood infections. Curr Opin Infect Dis.

[29] WHO, Roll Back Malaria (2005).

[30] Verrest L, Wilthagen EA, Beijnen JH, Huitema ADR, Dorlo TPC (2021). Influence of malnutrition on the pharmacokinetics of drugs used in the treatment of poverty-related diseases: a systematic review. Clin Pharmacokinet.

[31] Danquah I, Dietz E, Zanger P (2009). Reduced efficacy of intermittent preventive treatment of malaria in malnourished children. Antimicrob Agents Chemother.

[32] Coldiron ME, Von Seidlein L, Grais RF (2017). Seasonal malaria chemoprevention: successes and missed opportunities. Malar J.

[33] Balikagala B, Fukuda N, Ikeda M (2021). Evidence of artemisinin-resistant malaria in Africa. N Engl J Med.

[34] Straimer J, Gandhi P, Renner KC, Schmitt EK (2022). High prevalence of Plasmodium falciparum K13 mutations in Rwanda is associated with slow parasite clearance after treatment with artemether-lumefantrine. J Infect Dis.

