# Supplementary material for: Does acute malnutrition in young children increase the risk of treatment failure following artemisinin-based combination therapy? A WWARN individual patient data meta-analysis
Source: Lancet Glob Health. 2024 Mar 12;12(4):e631–40. doi: 10.1016/S2214-109X(24)00003-2 (PMC10951956; doi:10.1016/S2214-109X(24)00003-2)
Supplement: French translation of the abstract [file mmc1.pdf]

THE LANCET
Global Health

Supplementary appendix 1

This translation in French was submitted by the authors and we reproduce it as supplied. It has not been peer reviewed. *The Lancet*'s editorial processes have only been applied to the original in English, which should serve as reference for this manuscript.

Cette traduction en français a été proposée par les auteurs et nous l'avons reproduite telle quelle. Elle n'a pas été examinée par des pairs. Les processus éditoriaux du *Lancet* n'ont été appliqués qu'à l'original en anglais et c'est cette version qui doit servir de référence pour ce manuscrit.

Supplement to: WWARN ACT Malaria and Malnutrition Study Group. Does acute malnutrition in young children increase the risk of treatment failure following artemisinin-based combination therapy? A WWARN individual patient data meta-analysis. *Lancet Glob Health* 2024; **12:** e631–40.

Résumé

Contexte La répartition géographique, démographique et socio-économique du paludisme et de la malnutrition se chevauchent largement. On ne sait toujours pas si la malnutrition affecte l'efficacité des combinaisons thérapeutiques à base d'artémisinine (CTA) recommandées par l'OMS. Une revue systématique publiée antérieurement et explorant ce problème n'a pas été concluante car les données étaient rares et hétérogènes. Ces résultats suggèrent d'explorer d'autres approches méthodologiques telles qu'une méta-analyse des données individuelles des patients pour répondre à cette question. L'objectif de cette étude était de réaliser une telle méta-analyse pour évaluer l'effet de la malnutrition (émaciation et retard de croissance) sur le traitement par des CTA d'enfants de moins de 5 ans souffrant d'un paludisme à falciparum non compliqué.

Méthodes Nous avons effectué une méta-analyse des données individuelles de patients provenant d'études identifiées par une revue systématique de la littérature publiée entre 1980 et 2018 dans PubMed, Global Health et Cochrane Libraries (PROSPERO CRD42017056934) et une inspection des données d'études cliniques hébergées par le WorldWide Antimalarial Resistance Network (WWARN) mesurant l'efficacité des CTA incluant des enfants de moins de 5 ans atteints de paludisme à falciparum non compliqué. L'association entre la malnutrition aiguë (émaciation) ou chronique (retard de croissance) et le risque de recrudescence ajusté par PCR au 42e jour (c'est-à-dire un nouvel épisode de paludisme dû à la même souche de parasites) ou de réinfection après le traitement, a été étudiée à l'aide d'une régression de Cox, et selon la positivité pour le parasite au 2e jour, à l'aide d'une régression logistique.

Résultats Les données de 36 études ont été inclues dans cette analyse, dont 31 conduites en Afrique. Sur les 11301 enfants éligibles, provenant de 75 sites d'étude, 11,5 % souffraient d'émaciation (poids-taille Z score [WHZ] < -2) et 31,8 % de retard de croissance (taille-âge Z score [HAZ] < -2). La diminution du WHZ était associée à un risque accru de parasitémie positive au deuxième jour (odds ratio ajusté [AHR] 1,12, IC à 95% 1,05-1,18 par unité ; p=0,0002), d'échec du traitement ([AHR] 1,14, IC à 95% 1,02-1,26, p=0,016) et de réinfection après traitement (AHR 1,09, 1,04-1,13, p=0,0003). Les enfants présentant une émaciation modérée (WHZ -2 à -1) avaient également un risque de recrudescence plus élevé (AHR 1,85, 1,29-2,65, p=0,0008 vs WHZ ≥0). Le retard de croissance n'était pas associé à une efficacité réduite des CTA.

Interprétation Les enfants de moins de 5 ans souffrant de malnutrition aiguë et présentant un paludisme à falciparum non compliqué présentaient un risque plus élevé de délai dans l'élimination des parasites, d'échec du traitement par CTA et de réinfections. Les retards de croissance étaient plus fréquents, mais n'étaient pas associés à des changements dans l'efficacité des CTA. La malnutrition aiguë a un impact sur l'absorption et le métabolisme des médicaments. Il est urgent de mener d'autres études pour optimiser les doses de CTA chez les enfants souffrant de malnutrition aiguë.
