# Supplementary material for: Does acute malnutrition in young children increase the risk of treatment failure following artemisinin-based combination therapy? A WWARN individual patient data meta-analysis
Source: Lancet Glob Health. 2024 Mar 12;12(4):e631–40. doi: 10.1016/S2214-109X(24)00003-2 (PMC10951956; doi:10.1016/S2214-109X(24)00003-2)
Supplement: Supplementary appendix 2 [file mmc2.pdf]

THE LANCET
Global Health

Supplementary appendix 2

This appendix formed part of the original submission and has been peer reviewed. We post it as supplied by the authors.

Supplement to: WWARN ACT Malaria and Malnutrition Study Group. Does acute malnutrition in young children increase the risk of treatment failure following artemisinin-based combination therapy? A WWARN individual patient data meta-analysis. *Lancet Glob Health* 2024; **12:** e631–40.

Does acute malnutrition in young children increase the risk of treatment failure following artemisinin-based combination therapy: a WWARN individual patient data meta-analysis

Supplementary Material

1. Supplementary methods 2
1-1. Data Acquisition 2
1-2. Variable definitions 2
1-3. Definition of Endpoints 2
1-4. Model building strategy 3
1-5. Multiple imputations 3
1-6. Risk of bias assessment 4
2. Main Analysis Supplementary Tables 5
Supplementary Table 1. Characteristics of studies included in the IPD meta-analysis 5
Supplementary Table 2· Summary of treatment administered 8
Supplementary Table 3. Univariable analysis of parasite positivity on Day 2 9
Supplementary Table 4. Univariable analysis of risk of recrudescence. 10
Supplementary Table 5. Univariable analysis of risk of reinfection 12
Supplementary Table 6. Prophylactic effect of treatment over 42 days of follow-up. 13
Supplementary Table 7. Risk of bias assessment 15
3· Main Analysis Supplementary Figures 18
Supplementary Figure 1. Distribution of children's weight and height in individual studies. 18
Supplementary Figure 2. Forest plots of the association between a decrease in weight-for-height z-score and treatment outcomes in individual studies. 19
Supplementary Figure 3. Forest plots of the association between a decrease in height-for-weight z-score and treatment outcomes in individual studies. 20
Supplementary Figure 4. Map of study sites 21
Supplementary Figure 5. Venn diagram of anthropometric measures. 21
Supplementary Figure 6. Total mg/kg dose by WHZ-score. 22
4. Sensitivity analysis 23
4-1. Assessment of study-specific data and influence of individual cohorts 23
4-2. Subgroup analysis restricted to studies using at least three molecular markers for identification of treatment outcomes 25
4-3. Multiple imputations of age 26
5· Supplementary References 27
6· Members of WWARN ACT Malaria and Malnutrition Study Group 27

1. Supplementary methods

1-1. Data Acquisition

Investigators of studies satisfying the inclusion criteria were invited to participate in this IPD meta-analysis. Individual study protocols were available for all trials included, either from the publication or as a metafile submitted with the raw data. Individual patient data from eligible studies were previously shared with WWARN, collated and standardised using previously described methodology.[1] Analysis was conducted on data extracted as of 28 September 2020. Subsequently (last check April 2021) two additional studies were available with a total of 22 children treated with ASMQ or DP. Permission to use the data were not sought due to small number of eligible patients.

1-2. Variable definitions

The doses of artemisinin derivatives and the partner compounds received were calculated from the number of daily tablets administered to each patient. If the daily tablet counts were not available, doses were back-calculated using the dosing scheme available from study protocols.

The study sites were classified into 3 categories of Plasmodium falciparum malaria transmission intensity: low, medium and high malaria based on the Plasmodium falciparum parasite prevalence estimates PfPR obtained from the Malaria Atlas Project for specific location and year of study (for methodology see [2]). Study sites with a PfPR <0·10 were classified as low, with PfPR from 0·15 to <0·40 as moderate and with PfPR ≥0·40 as high transmission areas.[3]

Nutritional status was assessed with standardised age, weight, height and gender specific growth reference according to the WHO 2006 recommendations using igrowup Stata package 11. Anthropometric indicators include weight-for-age (WAZ), height-for-age (HAZ), and weight-for-height (WHZ). The nutritional status of a child were given as a Z-score and classified as underweight (WAZ<-2), stunted (HAZ<-2), or wasted (WHZ<-2) as defined in the WHO guidelines.[4, 5]

In patients with missing haemoglobin concentration, haematocrit was converted to haemoglobin using the following relationship[6]:

$$Hematocrit\ (ht) = 5 \cdot 62 + 2 \cdot 60\ \times Haemoglobin\ (hb)$$

Anaemia was defined as severe when haemoglobin concentration was below 7g/dl and as moderate if haemoglobin concentration was between 7-10g/dL.

1-3. Definition of Endpoints

a) Early Parasitological response

A pre-defined algorithm was used to estimate positivity status on days 2 or 3, if no observation of the blood film was recorded on that day as described in WWARN clinical Module DMSAP [1]. For studies with frequent or irregular sampling, a patient was classified as being positive on days 1, 2 and 3 after enrolment if the measurements within a window of ±2 hours of 24, 48 and 72 hours were positive.

b) Plasmodium falciparum recrudescence

Recrudescence was defined as recurrence of asexual Pf parasitaemia of the same genotype(s) that caused the original illness, due to incomplete clearance of asexual parasites after antimalarial treatment.

For the analysis of Plasmodium falciparum recrudescence, event was defined as PCR confirmed Pf recrudescence. Patients who had a new infection (of any species) were censored at the first day the positive parasitaemia was recorded.

Patients who had a reappearance of Pf parasites but the PCR result was not available were excluded from the analysis to avoid informative censoring.

c) *Plasmodium falciparum re-infection*

Reinfection was defined as a new Pf infection that follows a primary infection; distinguished from recrudescence by the parasite genotype, which is often (but not always) different from that which caused the initial infection.

For the analysis of Plasmodium falciparum re-infection, failure were defined as PCR confirmed Pf re-infection. Patients who had a new infection other than Pf species or Pf recrudescence were censored at the first day the positive parasitaemia was recorded. Patients who had a reappearance of Pf parasites but the PCR result was available were excluded from the analysis to avoid informative censoring.

1-4. Model building strategy

For all multivariable analyses the same approach was used. The WHZ score, age category and ACT treatment were kept in the model regardless of their statistical significance. The remaining covariates were investigated for inclusion in the model using a general strategy recommended by Collett.[7]

i) All possible risk factors were examined in a univariable analysis. The log-likelihood estimates $(-2 \times Log\hat{L})$ were compared against the null model to assess if any of the variables reduce its value at 5% level of statistical significance.
ii) All the variables identified in step (i) were fitted together in one model and variables which were not significant in the presence of other variables based on the results of the Wald test were identified.
iii) A likelihood ratio test was used to assess the impact of omitting variables identified in step (ii). If the omitted variable did not significantly impact the model log-likelihood, then they were dropped. Only variables which led to significant change in log-likelihood were retained.
iv) All variables excluded from step (i) were added to the model identified in step (iii) one by one to check if they provided any improvement to the model.
v) A final check of the model identified in step (iv) was carried out to ensure that none of the variables in the model could be omitted without significantly increasing the model log-likelihood, and none of the excluded variables significantly reduced the model log-likelihood.
vi) In the final model identified in v) interaction term between ACT and WHZ was added and likelihood ratio test was used to assess the improvement to the model.

The underlying assumption of proportional hazards was tested in the final model using Schenfoeld residuals and reported when violated. For variables which violated the assumption, interaction between this variable and time intervals (0-28, 28-42 days) or (0-21,21-28,28-42 days) were considered for inclusion in the final model.

Fractional polynomials[8] were used to adjust for the nonlinear relationship with continuous variables.

Interaction terms between different ACTs and nutritional covariates were explored to assess if the malnutrition effect is different for different treatments.

1-5. Multiple imputations

For studies with age measured in years for all or majority of children (>50%), multiple univariable imputation for such values were conducted using an interval regression imputation method (stata command*: mi impute intreg*[9]), assuming for a child with recorded age of x years that their true age is between x years 0 months and x years 11 months. All variables assessed in the multivariable models: weight, height, sex, study site. anaemia, fever, $\log_{10}$ parasitaemia, ACT, transmission intensity area were included as auxiliary variables in the imputation model. Outcome variables: parasite positivity on day 2, recrudescence by day 42, new infection by day 42, day of outcome, cumulative hazard (Nelson-Aalen estimator) for recrudescnce and for new infection were also included. Twenty datasets were imputed.

For each imputed dataset, anthropometric measures (WHZ, HAZ) were estimated using the imputed age and the final models were fitted. Model coefficients and standard errors were adjusted for the variability between imputations (stata command*: mi estimate)* according to the combination rules by Rubin.[10]

1-6. Risk of bias assessment

Risk of bias within studies was assessed based on: 1) study design (randomization, sequence generation, blinding); 2) accuracy in estimation of anthropometric indices and extent of the out-of-range values; and 3) the number and proportion of patients with (a) missing outcomes and (b) missing baseline covariates (age, weight, parasitaemia, temperature, haemoglobin).

In the sensitivity analysis, the final models, were refitted (a) with each study's data excluded, one at a time, and a coefficient of variation around the parameter estimates calculated; (b) in studies which used at least three molecular markers to distinguish recrudescence from the reinfection; (c) using multiple imputations for age in studies which have not provided age in months, or in which majority of children had age given as integer. Exclusion of studies would identify any influential studies, that is, studies with unusual results (due to variations in methodology, patient population, or other reasons) that affect the overall pooled analysis findings.

2. Main Analysis Supplementary Tables

Supplementary Table 1. Characteristics of studies included in the IPD meta-analysis

Study ID	Pubmed ID	Study Design	Location	Year	ACT	Duration of FU
1	20361381; 17884832	cohort	Gabon	2005	ASMQ	28
2	21740570	RCT	Cameroon, Cote d'Ivoire	2008-2009	AL	28
3	19128455	RCT	Mali	2005	ASAQ	28
4	27599612	RCT	Democratic Republic of Congo	2013-2014	AL, ASAQ	42
5	15837358	single arm	Kenya, Nigeria, Tanzania	2002-2003	AL	28
6	UNPUBLISHED	RCT	Niger	2011	AL, ASAQ	28
7	20689585; 17519410; 19841149	RCT	Uganda	2004-2008	AL, ASAQ	28
8	24354627	RCT	Senegal	2010-2012	AL,ASAQ	42
9	22458860	RCT	India	2007	ASAQ	28
10	19454000	single arm	India	2007	AL	28
11	24825870; 19877969; 21383095	RCT	Uganda	2007-2009	AL, DP	28
12	23270636	RCT	Cameroon, Cote d'Ivoire, Senegal	2007-2008	AL, ASAQ	28
13	22087077	RCT	Burkina Faso, Gabon, Mozambique, Nigeria, Rwanda, Uganda, Zambia	2007-2008	AL, DP	28
14	UNPUBLISHED		Liberia	2010-2011	ASAQ	28
15	22824059	single arm	Ethiopia	2008-2009	AL	42
16	19936217	RCT	Burkina Faso, Kenya, Mozambique, Uganda, Zambia	2005-2006	AL, DP	42
17	19187521	single arm	Kenya	2007	ASAQ	28
18	25108397	cohort	Mozambique	2011-2012	AL, ASAQ	28
19	16903879	RCT	Lao People’s Democratic Republic	2004	ASMQ, DP	42
20	UNPUBLISHED	RCT	Cote d' Ivoire	2012	AL, ASAQ	28
21	34205228	cohort	Mali	2009	AL	28
22	21838909	RCT	Senegal	2007-2008	AL, ASAQ	28
23	27430374	RCT	Burkina Faso, Kenya, Tanzania	2010-2013	AL, ASMQ	42
24	25436614	RCT	Uganda	2008	AL, ASAQ	42
25	17477865	RCT	Guinea	2004	ASAQ	28
26	27776521	single arm	Mali, Niger	2013-2015	AL	42
27	UNPUBLISHED	PK study	Ghana	2011-2012	ASAQ	42
28	19505304	RCT	Cameroon, Madagascar, Mali, Senegal	2006	AL, ASAQ	28
29	23866774	RCT	Liberia	2008-2009	AL, ASAQ	42
30	25075834	RCT	Democratic Republic of Congo	2013	AL	28
31	20065010	RCT	Senegal	2008	AL, ASMQ	28
32	25240962	single arm	Tanzania	2013	AL	28
33	25001306	RCT	Democratic Republic of Congo	2011-2012	AL, ASAQ, DP	42
34	27313266	cohort	Thailand	2003-2012	ASMQ	42
35	25549086	RCT	Papua New Guinea	2010-2012	AL	42
36	16163624	RCT	Tanzania	2002-2003	AL, ASAQ	42

Study ID	Malnutrition Exclusion criteria	N total included	Age Median	Min	Max	Underweight N evaluated	%	Stunted N evaluted	%	Wasted N evaluated	%
1	None	26	3·00	1·00	4·00	26	0·0	26	7·7	26	3·8
2	Severe malnutrition	34	3·00	2·00	4·00	33	3·0	34	26·5	28	3·6
3	None	131	3·00	0·50	4·91	131	8·4	131	13·7	131	8·4
4	weight-for-height below -3 Z-score and/or symmetrical oedemas involving at least the feet	249	2·58	0·50	4·83	249	22·9	247	44·1	249	4·4
5	weight for height <70% of the median NCHS/WHO reference	270	1·66	0·16	4·91	270	20·4	265	39·2	268	10·4
6	weight-to-height ratio <-3 z-score and / or oedema	81	3·00	0·25	4·50	80	16·3	77	27·3	80	7·5
7	weight-for-height or height-for-age z-score <-3	107	3·47	1·05	4·94	107	3·7	107	25·2	107	1·9
8	Severe malnutrition	11	3·00	1·00	4·00	11	18·2	11	9·1	10	20·0
9	None	13	4·00	0·58	4·00	13	30·8	13	61·5	13	0·0
10	None	3	3·00	3·00	4·00	3	100·0	2	100·0	3	33·3
11	None	271	0·83	0·31	2·67	271	15·5	271	29·5	271	3·0
12	weight-for-height <70% of the median NCHS/WHO reference value, with a symmetrical oedema involving the feet	193	3·00	0·91	4·00	192	13·5	190	24·2	191	12·6
13	weight for height <70% of the median NCHS/WHO	3,636	2·33	0·50	4·91	3636	22·1	3625	32·2	3624	12·9
14	weight- for-height is below -3 Standard Deviation or <70% of the median of the NCHS/WHO reference and/or oedema	102	2·54	0·66	4·83	102	14·7	101	21·8	102	11·8
15	severe malnutrition	47	3·00	1·00	4·50	47	23·4	46	28·3	47	23·4
16	weight for height <70% of the median NCHS/WHO reference	1,534	2·30	0·49	4·98	1534	15·6	1522	41·9	1527	3·9
17	malnutrition	90	2·58	0·50	4·91	90	4·4	90	22·2	89	4·5
18	wfh <70% of median NCHS/WHO reference	661	2·39	0·49	4·99	661	16·0	600	30·2	616	7·8
19	malnutrition	32	3·30	1·00	4·00	32	40·6	32	43·8	31	32·3
20	None	94	3·00	2·00	4·00	94	9·6	93	6·5	93	22·6
21	severe malnutrition	105	3·42	1·00	4·77	105	22·9	104	26·0	105	27·6
22	None	67	3·00	0·80	4·80	67	14·9	67	7·5	64	51·6
23	None	911	2·35	0·50	4·98	911	14·8	905	24·4	903	12·4
24	None	400	2·00	0·50	4·70	400	25·5	399	37·3	400	9·3
25	severe malnutrition MUAC <110mm, or oedema	3	2·91	0·91	3·00	3	100·0	3	33·3	3	100·0
26	None	389	1·83	0·50	4·83	389	50·4	389	32·1	388	42·8
27	None	25	1·00	0·50	4·00	22	9·1	25	0·0	18	16·7
28	None	251	3·00	0·90	4·80	251	22·7	250	27·2	247	20·7
29	weight-for-height below 70% of median NCHS/WHO reference and/or symmetrical oedemas involving at least the feet	295	3·06	0·71	5·00	295	12·5	294	32·7	295	2·7
30	None	35	3·29	0·67	4·83	35	22·9	34	32·4	34	2·9
31	weight-for-height below 70% of median NCHS/WHO reference and/or symmetrical oedemas involving at least the feet	32	3·00	1·00	4·00	32	12·5	32	6·3	32	28·1
32	growth standard is below –3 z-score, has symmetrical oedema involving at least the feet or has a mid-upper arm circumference < 110 mm)	56	3·28	1·00	4·96	56	7·1	55	3·6	55	20·0
33	Malnutrition	675	2·91	0·25	4·91	675	13·6	671	25·0	672	7·7
34	None	17	4·00	1·00	4·00	17	23·5	17	35·3	17	17·6
35	Malnutrition	67	3·08	1·08	4·91	67	26·9	66	33·3	66	13·6
36	None	388	2·33	0·41	4·91	388	18·6	377	44·0	384	7·8

Study ID	Median (Range)		Genotyping method	Number of patients with recurrence			Kaplan-Meier estimate (95% CI) at end FU	
	Weight	Height		RC	RI	no PCR	RC	RI
1	14.3(10.2-16.5)	97(76-105)	MSP-1 and MSP-2	0	0	0	1.00(.-.)	1.00(.-.)
2	14.5(11.0-28.0)	93(69-112)	MSP-1 and MSP-2	1	0	0	0.97(0.79-1.00)	1.00(.-.)
3	13.1(6.3-20.1)	93(63-115)	MSP-1 and MSP-2; CA1	5	51	4 (6·1)	0.95(0.89-0.98)	0.59(0.49-0.67)
4	11.0(6.1-17.0)	84(64-105)	MSP-1 and MSP-2, glurp	6	32	3 (7·3)	0.97(0.94-0.99)	0.86(0.81-0.90)
5	9.7(5.0-18.0)	79(51-111)	MSP-2	9	21	0	0.96(0.93-0.98)	0.92(0.88-0.95)
6	12.1(6.1-18.8)	88(48-117)	MSP-1 and MSP-2, glurp	1	7	1	0.99(0.90-1.00)	0.91(0.82-0.95)
7	14.0(10.0-20.0)	94(72-112)	MSP-1 and MSP-2	1	1	0	0.99(0.93-1.00)	0.99(0.93-1.00)
8	13.0(8.0-18.2)	100(78-113)	MSP-1 and MSP-2	0	0	0	1.00(.-.)	1.00(.-.)
9	13.0(7.2-22.0)	93(64-119)	MSP-1 and MSP-2, glurp	0	1	2	1.00(.-.)	0.91(0.51-0.99)
10	8.0(8.0-9.0)	79(70-81)	MSP-1 and MSP-2	0	0	0	1.00(.-.)	1.00(.-.)
11	8.0(5.2-13.5)	70(58-90)	MSP-1 and MSP-2; four miscrosatellite markers	0	32	1	1.00(.-.)	0.88(0.83-0.91)
12	13.4(6.0-22.0)	89(59-112)	MSP-1 and MSP-2	3	1	0	0.98(0.95-0.99)	0.99(0.96-1.00)
13	11.0(5.1-24.9)	84(54-122)	MSP-1 and MSP-2, glurp	86	457	62 (10·2)	0.97(0.97-0.98)	0.86(0.85-0.87)
14	12.0(6.3-18.7)	88(69-105)	MSP-1 and MSP-2	0	10	3 (23·1)	1.00(.-.)	0.89(0.81-0.94)
15	12.0(7.0-16.0)	92(60-115)	MSP-1 and MSP-2, glurp	3	2	0	0.93(0.80-0.98)	0.95(0.82-0.99)
16	11.0(6.0-25.5)	83(53-117)	MSP-1 and MSP-2, glurp	70	230	30 (9·1)	0.95(0.94-0.96)	0.84(0.82-0.85)
17	12.0(6.6-18.0)	86(64-114)	MSP-2	9	17	1	0.89(0.80-0.94)	0.80(0.69-0.87)
18	11.7(5.0-23.1)	83(43-123)	MSP-1 and MSP-2, glurp	15	24	1	0.98(0.96-0.98)	0.96(0.94-0.97)
19	11.5(6.5-16.5)	91(64-110)	MSP-1 and MSP-2, glurp	0	2	0	1.00(.-.)	0.94(0.77-0.98)
20	13.7(9.0-21.0)	99(73-113)	MSP-1 MSP-2	0	1	3	.(.-.)	.(.-.)
21	13.0(9.0-18.0)	95(76-110)	MSP-1 and MSP-2; CA1	5	23	2	0.94(0.87-0.98)	0.77(0.67-0.84)
22	11.8(7.6-19.1)	96(70-122)	MSP-1 MSP-2	0	3	6 (66·7)	1.00(.-.)	0.95(0.84-0.98)
23	11.2(5.2-23.0)	86(60-114)	MSP-1 and MSP-2, glurp	20	266	39 (12·0)	0.97(0.95-0.98)	0.66(0.63-0.70)
24	10.0(6.0-18.0)	82(62-107)	MSP-1 and MSP-2, glurp	6	258	11 (4·0)	0.98(0.96-0.99)	0.32(0.27-0.37)
25	8.8(7.0-9.8)	84(72-93)	MSP-1 and MSP-2, glurp	0	0	0	1.00(.-.)	1.00(.-.)
26	8.8(5.0-17.6)	79(63-112)	MSP-2; CA1and TA87	4	94	1	0.99(0.97-1.00)	0.75(0.70-0.79)
27	10.2(5.5-28.0)	80(65-101)	MSP-1 and MSP-2, glurp	1	1	1	0.96(0.74-0.99)	0.96(0.74-0.99)
28	12.0(10.0-22.0)	92(69-130)	MSP-1 and MSP-2; CA1	6	30	0	0.97(0.94-0.99)	0.87(0.82-0.91)
29	12.9(7.0-20.0)	91(67-115)	MSP-1 and MSP-2, glurp	9	109	8 (6·3)	0.96(0.92-0.98)	0.61(0.55-0.66)
30	12.0(7.0-19.0)	92(69-111)	MSP-1 and MSP-2, glurp	1	9	0	0.97(0.81-1.00)	0.74(0.56-0.85)
31	13.1(10.0-18.9)	97(77-109)	MSP-1 and MSP-2	1	0	0	0.97(0.80-1.00)	1.00(.-.)
32	13.3(8.0-19.5)	98(73-140)	MSP-1 and MSP-2, glurp	0	11	2	1.00(.-.)	0.78(0.64-0.87)
33	12.0(5.3-20.1)	89(61-118)	MSP-1 and MSP-2, glurp	30	106	6 (4·2)	0.95(0.93-0.96)	0.83(0.80-0.86)
34	13.0(10.0-21.0)	94(72-104)	MSP-1 and MSP-2, glurp	2	0	3	0.86(0.54-0.96)	1.00(.-.)
35	12.0(6.5-16.0)	91(37-107)	MSP-1 MSP-2	1	2	2	0.98(0.87-1.00)	0.96(0.87-0.99)
36	11.0(6.0-20.0)	83(37-108)	MSP-2	24	89	8 (6·6)	0.93(0.90-0.95)	0.75(0.71-0.80)

* RC= Recrudescence RI = Reinfection, evaluated at the end of study follow-up (28 or 42 days)

Supplementary Table 2· Summary of treatment administered

Supplementary Table 2· Summary of treatment administered

Treatment	N [%]	Artemisinin Derivative (AD) N with dose	Artemisinin Derivative (AD) Median [Range]	Artemisinin Derivative (AD) Underdosed[1] N [%]	Partner Drug (PD) N with dose	Partner Drug (PD) Median [Range]	Partner Drug (PD) Underdosed[2] N [%]
AL	5015 [44·4]	4887	11·4 [1·4 -86·3]	74 [1·5]	5012	68·6 [8·3 - 518·0]	1533 [30·6]
ASAQ - FDC	2321 [20·5]	2321	12·5 [2·2 - 33·3]	9 [0·4]	2321	33·8 [6·0 - 90·0]	23 [1·0]
ASAQ - nFDC	583 [5·2]	583	12·1 [7·1-21·3]	0 [0]	583	30·0 [20·3 - 64·3]	22 [3·8]
ASMQ	531 [4·7]	516	11·5 [6·3 - 26·3]	0 [0]	516	24·8 [12·5 - 52·6]	2 [0·4]
DP	2851 [25·2]	2850	6·9 [1·7 - 19·8]	1609 [56·5]	2850	55·2 [13·2-158·0]	1609 [56·5]
All ACTs	11301 [100]	11157	NA	1692 [15·2]	11282	NA	3189 [28·3]

[1] Below minimum total dose defined based on WHO guidelines as <5mg/kg for AL, <7·5 mg/kg for DP and children<25kg and <6mg/kg for all other regimens

[2] Below minimum total dose defined based on WHO guidelines as < 29 mg/kg for lumefantrine, <22·5mg/kg for AQ, <15mg/kg for MQ, <48mg/kg for DP in patients weighing >=25kg and <60mg/kg for DP in patient weight <25kg

AL Artemether-lumefantrine; ASAQ Artesunate-Amodiaquine; ASMQ Artesunate-Mefloquine; DP Dihydroartemisinin-piperaquine; (n)FDC (non) Fixed Dose Combination; ACTs Artemisinin-based Combination Therapies

Supplementary Table 3. Univariable analysis of parasite positivity on Day 2

Supplementary Table 3. Univariable analysis of parasite positivity on Day 2· Logistic regression models with random intercept for study-site were fitted.

Parameter		N included	N positive	% positive	OR (95% CI)	P-value
ACT	AL	4791	472	9·9	1·23 (0·99 - 1·51)	0·060
	ASAQ - FDC	2309	179	7·8	0·62 (0·48 - 0·80)	<0·001
	ASAQ - nFDC	581	57	9·8	1·42 (0·75 - 2·69)	0·288
	ASMQ	525	30	5·7	0·96 (0·56 - 1·65)	0·889
	DP	2842	284	10·0	1	
Age (years)		11048	1022	9·3	1·00 (0·94 - 1·06)	0·886
Age group	< 1 year	1132	87	7·7	0·92 (0·68 - 1·24)	0·572
	1 year	2905	247	8·5	1·05 (0·84 - 1·32)	0·671
	2 years	2768	283	10·2	1·16 (0·93 - 1·44)	0·182
	3 years	2317	222	9·6	1·01 (0·80 - 1·27)	0·942
	4 years	1926	183	9·5	1	
Sex:	Male	5738	536	9·2	1·10 (0·95 - 1·27)	0·195
	Female	5310	486	9·3	1	
Haemoglobin (g/dL)		10858	997	9·2	0·98 (0·93 - 1·02)	0·258
Anaemia[1]	Severe	844	94	11·1	1·30 (1·00 - 1·69)	0·048
	Moderate	6190	552	8·9	1·00 (0·86 - 1·17)	0·993
	No anaemia	3824	351	9·2	1	
Temperature (^{o}C)		10939	1020	9·3	1·30 (1·21 - 1·38)	<0·001
Fever [2]	Yes	6991	762	10·9	1·53 (1·30 - 1·82)	<0·001
	No	4055	260	6·4	1	
Log_{10} parasitaemia		11048	1022	9·3	2·26 (1·95 - 2·62)	<0·001
Hyperparasiteamia[3]	Yes	1596	255	16·0	2·24 (1·88 - 2·68)	<0·001
	No	9452	767	8·1	1	
Presence of gametocytes	Yes	980	43	4·4	0·71 (0·50 - 1·02)	0·065
	No	7854	741	9·4	1	
Region:	Asia[4]	124	17	13·7	2·57 (0·63 - 10·51)	0·188
	Central Africa	1329	71	5·3	0·52 (0·16 - 1·73)	0·284
	Eastern Africa	6108	613	10·0	0·71 (0·33 - 1·53)	0·381
	Western Africa	3487	321	9·2	1	
TIA[5]	Low	4596	392	8·5	0·40 (0·24 - 0·66)	<0·001
	Moderate	2283	338	14·8	0·53 (0·35 - 0·81)	0·003
	High	4169	292	7·0	1	
Weight-for-age z-score (WAZ)		11042	1021	9·2	0·92 (0·86 - 0·98)	0·010
Underweight[6]	WAZ<-1	5453	466	8·6	1·09 (0·94 - 1·26)	0·262
	WAZ<-2	2067	165	8·0	1·10 (0·91 - 1·34)	0·337

WAZ<-3	516	35	6·8	1·00 (0·68 - 1·47)	0·986
Weight-for-height z-score (WHZ)	10938	1009	9·2	0·90 (0·85 - 0·95)	<0·001
Wasting[6] WHZ<-1	3337	277	8·3	1·19 (1·01 - 1·40)	0·043
WHZ<-2	1187	96	8·1	1·21 (0·94 - 1·55)	0·135
WHZ<-3	319	30	9·4	1·34 (0·88 - 2·06)	0·174
Height-for-age z-score (HAZ)	10919	1007	9·2	1·03 (0·98 - 1·08)	0·288
Stunting[6] HAZ<-1	6593	609	9·2	0·98 (0·84 - 1·14)	0·798
HAZ<-2	3483	332	9·5	0·98 (0·84 - 1·16)	0·847
HAZ<-3	1310	136	10·4	0·89 (0·71 - 1·11)	0·291

[1]Severe anaemia defined as hb<7g/dL, moderate anaemia as hb between 7 and 10g/dL; [2] fever defined as temperature >37·5°C or history of fever; [3] defined as parasitaemia>10^5/µL; [4] includes children from India, Lao PDR, Papua New Guinea and Thailand;[5]TIA=Transmission Intensity Areas; [6]Compared to the rest of the children, for example WHZ<-1 is compared to WHZ≥-1·

Supplementary Table 4. Univariable analysis of risk of recrudescence.

Supplementary Table 4. Univariable analysis of risk of recrudescence by day 42· Cox regression model with shared frailty for study-site was fitted.

Parameter	N included	N positive	% positive	HR (95% CI)	P-value
ACT AL	4911	148	3·0	1·38 (1·03 - 1·85)	0·033
ASAQ - FDC	2283	43	1·9	0·86 (0·57 - 1·28)	0·448
ASAQ - nFDC	573	31	5·4	2·55 (1·45 - 4·49)	0·001
ASMQ	506	11	2·2	0·90 (0·44 - 1·85)	0·774
DP	2820	86	3·0	1	
Age (years)	11093	319	2·9	0·95 (0·86 - 1·04)	0·257
Age group < 1 year	1154	27	2·3	1·15 (0·70 - 1·90)	0·583
1 year	2950	92	3·1	1·50 (1·02 - 2·21)	0·038
2 years	2761	100	3·6	1·74 (1·19 - 2·53)	0·004
3 years	2315	62	2·7	1·31 (0·87 – 2·00)	0·191
4 years	1913	38	2·0	1	
Sex: Male	5761	173	3·0	1·09 (0·87 - 1·36)	0·456
Female	5332	146	2·7	1	
Haemoglobin (g/dL)	10900	310	2·8	0·91 (0·85 - 0·98)	0·011
Anaemia[1] Severe	858	40	4·7	1·72 (1·17-2·53)	0·006
Moderate	6229	181	2·9	1·18 (0·91 – 1·53)	0·217
No anaemia	3813	89	1·3	1	
Temperature (°C)	10977	317	2·9	0·97 (0·88 - 1·07)	0·549
Fever [2] Yes	7059	186	2·6	0·90 (0·71 - 1·14)	0·373
No	4027	133	3·3	1	

Log_{10} Parasitaemia (/µL)		11093	319	2·9	1·31 (1·06 - 1·61)	0·011
Hyperparasiteamia[3]	Yes	1575	52	3·3	1·23 (0·90 - 1·66)	0·192
	No	9518	267	2·8	1	
Presence of gametocytes	Yes	971	43	4·4	1·34 (0·96 - 1·89)	0·089
	No	7711	228	3·0	1	
Region	Asia[4]	128	3	2·3	0·80 (0·20 - 3·24)	0·750
	Central Africa	1324	41	3·1	0·74 (0·35 - 1·56)	0·431
	Eastern Africa	5997	164	2·7	0·99 (0·63 - 1·56)	0·979
	Western Africa	3644	111	3·0	1	
TIA[5]	Low	4538	108	2·4	0·68 (0·44 - 1·07)	0·096
	Moderate	2260	80	3·5	1·37 (0·86 - 2·18)	0·185
	High	4295	131	3·1	1	
Weight-for-age z-score (WAZ)		11087	318	2·9	0·94 (0·85 - 1·04)	0·239
Underweight[6]	WAZ<-1	5528	168	3·0	1·06 (0·84 - 1·32)	0·636
	WAZ<-2	2149	63	2·9	1·04 (0·78 - 1·38)	0·784
	WAZ<-3	571	11	1·9	0·71 (0·39 - 1·31)	0·279
Weight-for-height z-score (WHZ)		10982	314	2·9	0·93 (0·85 - 1·02)	0·126
Wasting[6]	WHZ<-1	3422	115	3·4	1·34 (1·05 - 1·70)	0·018
	WHZ<-2	1256	34	2·7	1·03 (0·71 - 1·48)	0·887
	WHZ<-3	377	6	1·6	0·69 (0·30 - 1·57)	0·375
Height-for-age z-score (HAZ)		10965	315	2·9	1·00 (0·92 - 1·07)	0·900
Stunting[6]	HAZ<-1	6639	204	3·0	1·12 (0·88 - 1·42)	0·355
	HAZ<-2	3495	101	2·9	0·96 (0·75 - 1·22)	0·706
	HAZ<-3	1296	36	2·8	0·91 (0·64 - 1·30)	0·611

[1]Severe anaemia defined as hb<7g/dL, moderate anaemia as hb between 7 and 10g/dL; [2] fever defined as temperature >37·5°C or history of fever; [3]defined as parasitaemia $>10^5$/µL; [4] includes children from India, Lao PDR, Papua New Guinea and Thailand;[5]TIA=Transmission Intensity Areas; [6]compared to the rest of the children, for example WHZ<-1 is compared to WHZ≥-1·

Supplementary Table 5. Univariable analysis of risk of reinfection

Supplementary Table 5. Univariable analysis of risk of reinfection by day 42· Cox regression model with shared frailty for study-site was fitted.

Parameter		N included	N positive	% positive	HR (95% CI)	P-value
ACT	AL	4910	984	20·0	2·80 (2·39 - 3·29)	<0·001
	ASAQ - FDC	2283	414	18·1	2·37 (1·97 - 2·86)	<0·001
	ASAQ - nFDC	573	130	22·7	5·87 (3·88 - 8·90)	<0·001
	ASMQ	506	129	25·5	2·28 (1·72 - 3·02)	<0·001
	DP	2820	233	8·3	1	
Age (years)		11092	1890	17·0	1·05 (1·01 - 1·09)	0·014
Age group	< 1 year	1154	163	14·1	0·70 (0·57 - 0·85)	<0·001
	1 year	2950	542	18·4	0·90 (0·77 - 1·04)	0·146
	2 years	2761	516	18·7	1·03 (0·89 - 1·19)	0·710
	3 years	2314	376	16·2	0·98 (0·84 - 1·14)	0·749
	4 years	1913	293	15·3	1	
Sex:	Male	5761	1024	17·8	1·10 (1·01 - 1·21)	0·033
	Female	5331	866	16·2	1	
Haemoglobin (g/dL)		10899	1874	17·2	0·97 (0·94 – 1·00)	0·038
Anaemia[1]	Severe	858	186	21·7	1·19 (1·00-1·41)	0·045
	Moderate	6228	1119	18·0	1·05 (0·949-1·17)	0·320
	No anaemia	3813	569	14·9	1	
Temperature (C^o)		10976	1885	17·2	1·01 (0·97 - 1·05)	0·685
Fever[2]:	Yes	7058	1156	16·4	0·98 (0·89 - 1·09)	0·710
	No	4027	730	18·1	1	
Log_{10} parasitaemia (/µL)		11092	1890	17·0	1·20 (1·10 - 1·31)	<0·001
Hyperparasiteamia[3]	Yes	1574	251	16·0	1·10 (0·96 - 1·26)	0·187
	No	9518	1639	17·2	1	
Presence of gametocytes.	Yes	971	235	24·2	1·18 (1·02 - 1·37)	0·026
	No	7710	1330	17·3	1	
Region:	Asia[4]	128	5	3·9	0·18 (0·05 - 0·68)	0·012
	Central Africa	1324	154	11·6	0·48 (0·22 - 1·01)	0·053
	Eastern Africa	5996	907	15·1	0·74 (0·46 - 1·17)	0·192
	Western Africa	3644	824	22·6	1	
TIA[5]	Low	4537	475	10·5	0·37 (0·24 - 0·56)	<0·001
	Moderate	2260	315	13·9	0·76 (0·50 - 1·15)	0·186
	High	4295	1100	25·6	1	

Weight-for-age z-score (WAZ)	11086	1890	17·0	0·96 (0·92 - 1·00)	0·054
Underweight[6] WAZ<-1	5527	1067	19·3	1·13 (1·03 - 1·24)	0·009
WAZ<-2	2148	430	20·0	1·08 (0·97 - 1·21)	0·176
WAZ<-3	571	95	16·6	0·86 (0·69 - 1·06)	0·147
Weight-for-height z-score (WHZ)	10981	1880	17·1	0·96 (0·92 – 1·00)	0·027
Wasting[6] WHZ<-1	3421	680	20·0	1·09 (0·99 - 1·21)	0·083
WHZ<-2	1256	278	22·1	1·26 (1·10 - 1·45)	0·001
WHZ<-3	377	75	19·9	1·09 (0·85 - 1·39)	0·511
Height-for-age z-score (HAZ)	10964	1880	17·1	1·00 (0·96 - 1·03)	0·797
Stunting[6] HAZ<-1	6638	1145	17·3	0·96 (0·88 - 1·06)	0·442
HAZ<-2	3494	603	17·3	1·03 (0·93 - 1·14)	0·551
HAZ<-3	1296	208	16·1	1·06 (0·91 - 1·23)	0·444

[1]Severe anaemia defined as hb<7g/dL, moderate anaemia as hb between 7 and 10g/dL; [2] fever defined as temperature >37·5ºC or history of fever; [3] defined as parasitaemia >10^5/µL; [4] includes children from India, Lao PDR, Papua New Guinea and Thailand; [5]TIA=Transmission Intensity Areas; [6]Compared to the rest of the children, for example WHZ<-1 is compared to WHZ≥-1·

Supplementary Table 6. Prophylactic effect of treatment over 42 days of follow-up.

Supplementary Table 6. Prophylactic effect of treatment over 42 days of follow-up. Results come from multivariable Cox regression model with shared frailty for study site, and interaction terms with time intervals (0-21, 21-28, 28-42) for variables which proportional hazard assumption was not satisfied.

Parameter	Time Interval (days)	AHR (95%CI)	p-value
Age group <1 year	0-42	0·65 (0·53 - 0·79)	<0·001
1 year	0-42	0·85 (0·73 - 0·99)	0·033
2 years	0-42	0·99 (0·85 - 1·15)	0·911
3 years	0-42	0·97 (0·828 - 1·13)	0·674
4 years	0-42	1	
WHZ-score	0-28	1·08 (1·03-1·13)	0·001
	28-42	0·94 (0·88-1·01)	0·081
Log_{10} parasitaemia (/µL)	0-21	1·52 (1·29 - 1·78)	<0·001
	21-42	1·11 (1·00 - 1·23)	0·059
ACT AL	0-42	1	
ASAQ-FDC	0-42	0·85 (0·74 - 0·97)	0·017
ASAQ-nFDC	0-21	2·80 (1·78 - 4·41)	<0·001
	21-42	1·80 (1·19 - 2·72)	0·005
ASMQ	0-21	0·25 (0·13 - 0·46)	<0·001
	21-42	1·07 (0·84 - 1·37)	0·578
DP	0-21	0·12 (0·08 - 0·19)	<0·001
	21-28	0·38 (0·30 - 0·48)	<0·001
	28-42	0·56 (0·44 - 0·71)	<0·001
Gender: Male	0-42	1·10 (1·00 - 1·20)	0·049

Female	0-42	1	
TIA[1]: High	0-42	2·86 (1·85 - 4·43)	<0·001
Moderate	0-42	2·01 (1·53 - 2·64)	<0·001
Low	0-42	1	
Anemia[2]: No	0-42	1	
Moderate	0-42	1·09 (0·98 - 1·21)	0·136
Severe	0-42	1·27 (1·07 - 1·52)	0·007

[1]TIA=Transmission Intensity Areas; [2]Severe anaemia defined as hb<7g/dL, moderate anaemia as hb between 7 and 10g/dL.

Supplementary Table 7. Risk of bias assessment

Supplementary Table 7. Risk of bias assessment. Part I. NI = No Information available n/a = not applicable

Study ID	Pubmed ID	Sample size	N Arms	Randomisation	Concealment of Treatment	Sequence Generation	Treatment Blinding	Treatment Supervision	PCR genotyping method
1	20361381; 17884832	Yes	1	No	n/a	n/a	n/a	full	MSP-1 and MSP-2
2	21740570	Yes	2	Yes	Opaque sealed envelopes	computer generated	open-label	full	MSP-1 and MSP-2
3	19128455	NI	3	Yes	NI	NI	patient blinded	full	MSP-1 and MSP-2; CA1
4	27599612	Yes	2	Yes	NI	NI	open-label	full	MSP-1 and MSP-2, glurp
5	15837358	Yes	1	No	n/a	n/a	n/a	full	MSP-2
6	UNPUBLISHED	NI	2	Yes	NI	randomization list	investigator blinded	full	MSP-1 and MSP-2, glurp
7	20689585; 17519410; 19841149	Yes	3	Yes	NI	NI	investigator blinded	partial (first daily dose only)	MSP-1 and MSP-2
8	24354627	Yes	3	Yes	NI	NI	open-label	full	MSP-1 and MSP-2
9	22458860	Yes	2	Yes	Opaque sealed envelopes	randomization list	open-label	full	MSP-1 and MSP-2, glurp
10	19454000	NI	1	No	n/a	n/a	n/a	full	MSP-1 and MSP-2
11	24825870; 19877969; 21383095	No	2	Yes	NI	computer generated	open-label	Partial (first daily dose only)	MSP-1 and MSP-2; four microsatellite markers
12	23270636	Yes	2	Yes	NI	NI	open-label	full	MSP-1 and MSP-2
13	22087077	Yes	4	Yes	Opaque sealed envelopes	independent organisation	open-label	full	MSP-1 and MSP-2, glurp
14	UNPUBLISHED	Yes	1	No	n/a	n/a	n/a	full	MSP-1 and MSP-2
15	22824059	Yes	1	No	n/a	n/a	n/a	partial (first dose only)	MSP-1 and MSP-2, glurp
16	19936217	Yes	2	Yes	Opaque sealed envelopes	independent organisation	open-label	full	MSP-1 and MSP-2, glurp
17	19187521	Yes	1	No	n/a	n/a	n/a	full	MSP-2
18	25108397	Yes	2	No	n/a	n/a	open-label	full	MSP-1 and MSP-2, glurp
19	16903879	NI	2	Yes	Opaque sealed envelopes	NI	open-label	full	MSP-1 and MSP-2, glurp
20	UNPUBLISHED	Yes	2	Yes	Opaque sealed envelopes	computer generated	open-label	full	MSP-1 MSP-2
21	UNPUBLISHED	NI	1	No	n/a	n/a	n/a	Partial (first daily dose only)	MSP-1 and MSP-2; CA1
22	21838909	NI	2	Yes	NI	NI	open-label	full	MSP-1 MSP-2

23	27430374	Yes	2	Yes	Opaque sealed envelopes	computer generated	laboratory blinded	full	MSP-1 and MSP-2, glurp
24	25436614	Yes	2	Yes	Opaque sealed envelopes	computer generated	open-label	full	MSP-1 and MSP-2, glurp
25	17477865	Yes	2	Yes	Without concealment	NI	open-label	full	MSP-1 and MSP-2, glurp
26	27776521	Yes	1	No	n/a	n/a	n/a	full	MSP-2; CA1and TA87
27	33407454	Yes	1	No	n/a	n/a	n/a	full	MSP-1 and MSP-2, glurp
28	19505304	Yes	3	Yes	NI	randomization list	investigator blinded	full	MSP-1 and MSP-2; CA1
29	23866774	Yes	2	Yes	Opaque sealed envelopes	computer-generated	open-label	full	MSP-1 and MSP-2, glurp
30	25075834	Yes	4	Yes	Opaque sealed envelopes	NI	open-label	full	MSP-1 and MSP-2, glurp
31	20065010	Yes	2	Yes	NI	NI	open-label	full	MSP-1 and MSP-2
32	25240962	Yes	1	No	n/a	n/a	n/a	full	MSP-1 and MSP-2, glurp
33	25001306	Yes	3	Yes	Opaque sealed envelopes	computer generated	open-label	full	MSP-1 and MSP-2, glurp
34	27313266	NI	1	No	n/a	n/a	n/a	full	MSP-1 and MSP-2, glurp
35	25549086	Yes	4	Yes	Opaque sealed envelopes	computer generated	open-label	partial (first daily dose only)	MSP-1 MSP-2
36	16163624	Yes	2	Yes	NI	NI	open-label	full	MSP-2

Supplementary Table 7. Risk of bias assessment. Part II

Study ID	Pubmed ID	% with missing data	%lost before Day 28	% lost before Day 42	N with RC	N with NI	N with no PCR	% Day2 missing	WFH invalid
1	20361381; 17884832	0·0	0·0		0	0	0	0·0	0·0
2	21740570	0·0	8·8		1	0	0	0·0	17·6
3	19128455	0·0	1·5		5	51	4 (6·1)	0·0	0·0
4	27599612	9·1	5·2	6·4	6	32	3 (7·3)	0·0	0·0
5	15837358	0·4	1·5		9	21	0	1·5	0·7
6	UNPUBLISHED	10·0	6·2		1	7	1	1·2	1·2
7	20689585; 17519410; 19841149	3·6	7·5		1	1	0	1·9	0·0
8	24354627	21·4	0·0	18·2	0	0	0	0·0	9·1
9	22458860	0·0	0·0		0	1	2	0·0	0·0
10	19454000	81·3	0·0		0	0	0	0·0	0·0
11	24825870; 19877969; 21383095	6·9	1·5		0	32	1	0·0	0·0
12	23270636	21·2	0·0		3	1	0	0·5	1·0
13	22087077	1·7	4·6		86	457	62 (10·2)	0·4	0·3
14	UNPUBLISHED	0·0	3·9		0	10	3 (23·1)	0·0	0·0
15	22824059	7·8	6·4	8·5	3	2	0	0·0	0·0
16	19936217	0·6	3·0	4·4	70	230	30 (9·1)	0·1	0·5
17	19187521	0·0	3·3		9	17	1	0·0	1·1
18	25108397	3·2	8·5		15	24	1	0·0	6·8
19	16903879	22·0	0·0	3·1	0	2	0	15·6	3·1
20	UNPUBLISHED	6·0	2·1		0	1	3	2·1	1·1
21	UNPUBLISHED	2·8	2·9		5	23	2	0·0	0·0
22	21838909	0·0	1·5		0	3	6 (66·7)	0·0	4·5
23	27430374	3·1	5·6	7·7	20	266	39 (12·0)	0·0	0·9
24	25436614	3·1	0·8	1·0	6	258	11 (4·0)	0·0	0·0
25	17477865	97·3	0·0		0	0	0	0·0	0·0
26	27776521	2·0	1·8	3·3	4	94	1	7·5	0·3
27	33407454	30·6	4·0	8·0	1	1	1	4·0	28·0
28	19505304	12·8	2·0		6	30	0	0·0	1·6
29	23866774	0·3	1·4	2·7	9	109	8 (6·3)	0·0	0·0
30	25075834	0·0	0·0		1	9	0	0·0	2·9
31	20065010	68·6	0·0		1	0	0	0·0	0·0
32	25240962	0·0	7·1		0	11	2	3·6	1·8
33	25001306	0·7	3·7	6·7	30	106	6 (4·2)	0·1	0·4
34	27313266	5·6	23·5	23·5	2	0	3	17·6	0·0
35	25549086	10·7	6·0	17·9	1	2	2	0·0	1·5
36	16163624	2·5	1·0	1·8	24	89	8 (6·6)	0·5	1·0

3· Main Analysis Supplementary Figures

Supplementary Figure 1. Distribution of children's weight and height in individual studies.
Number above each panel is the study id.

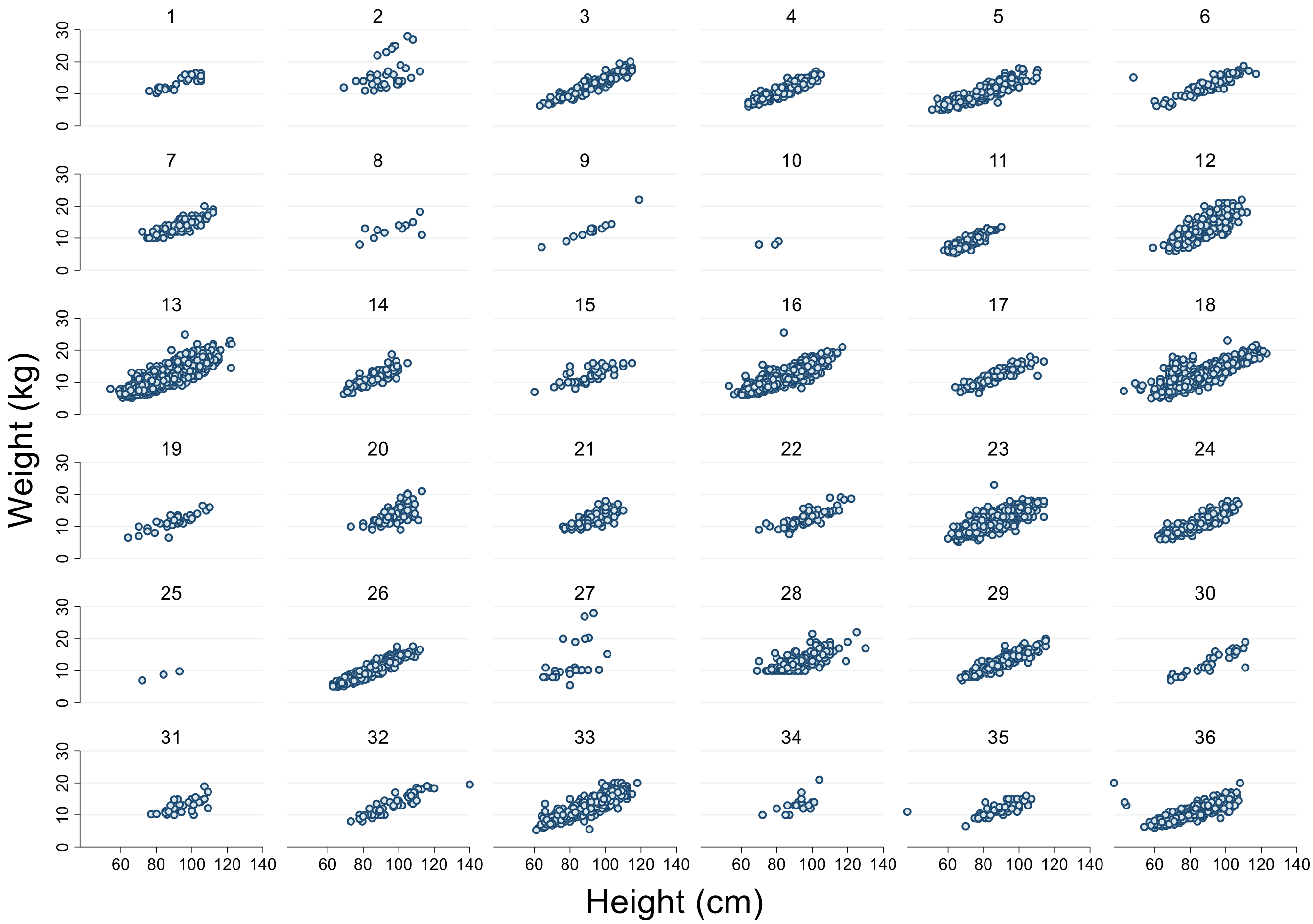

Supplementary Figure 2. Forest plots of the association between a decrease in weight-for-height z-score and treatment outcomes in individual studies.

Unadjusted estimates are presented due to small numbers. For studies omitted in the plots, data were too sparse so the model could not be fitted.

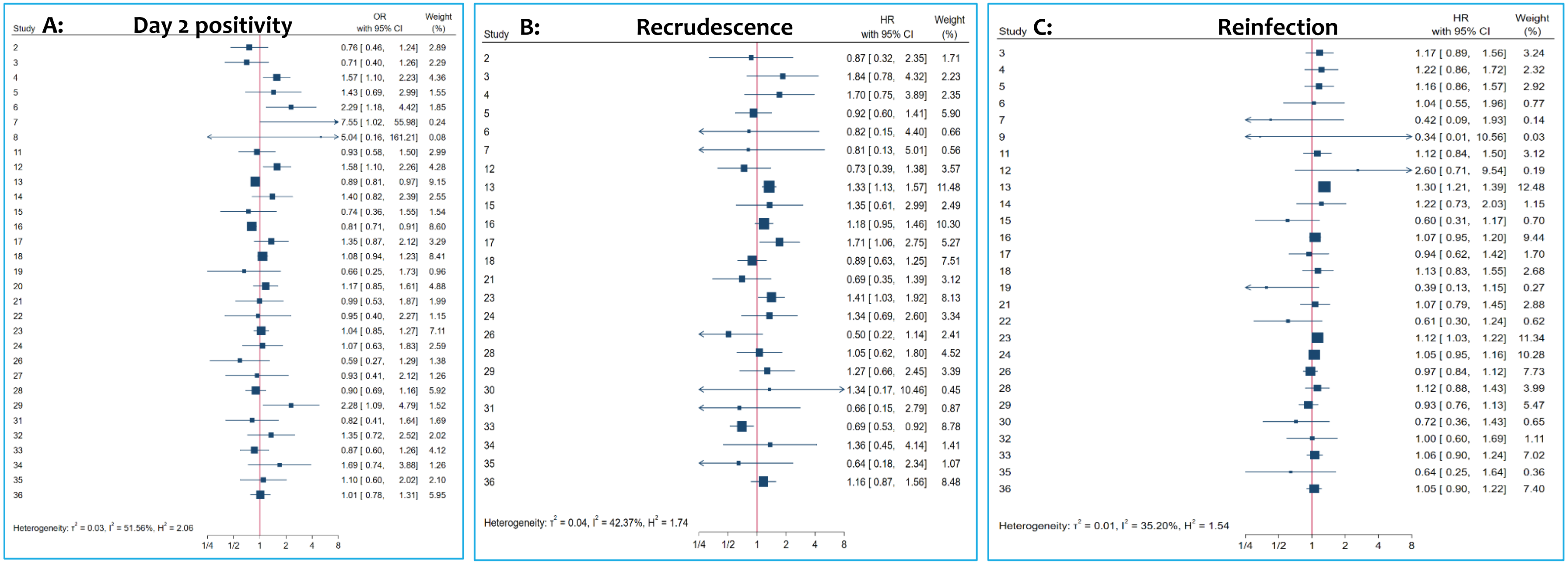

Supplementary Figure 3. Forest plots of the association between a decrease in height-for-weight z-score and treatment outcomes in individual studies.

Unadjusted estimates are presented due to small numbers. For studies omitted in the plots, data were too sparse so the model could not be fitted.

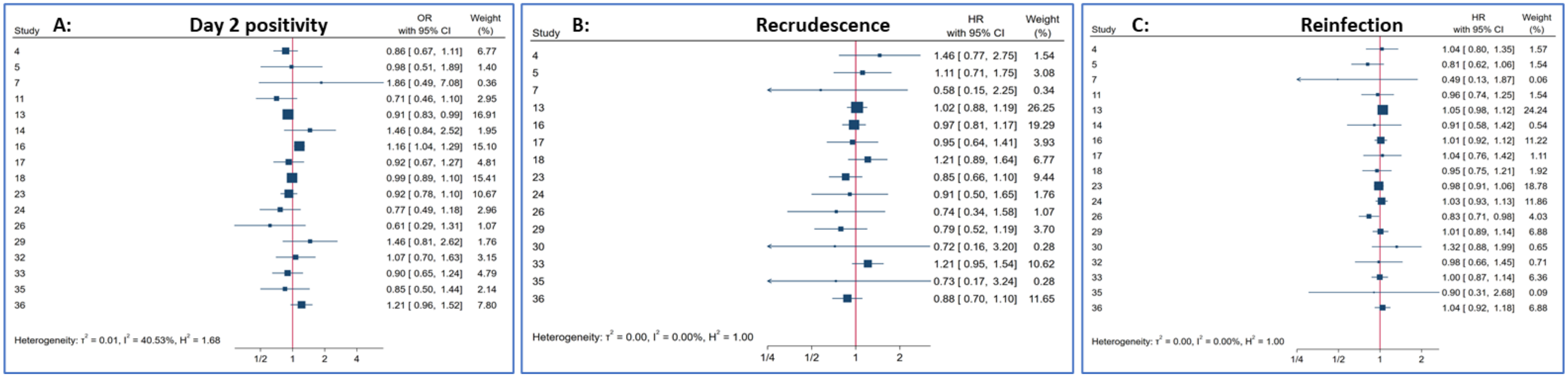

Supplementary Figure 4. Map of study sites

Supplementary Figure 4. Map of study sites

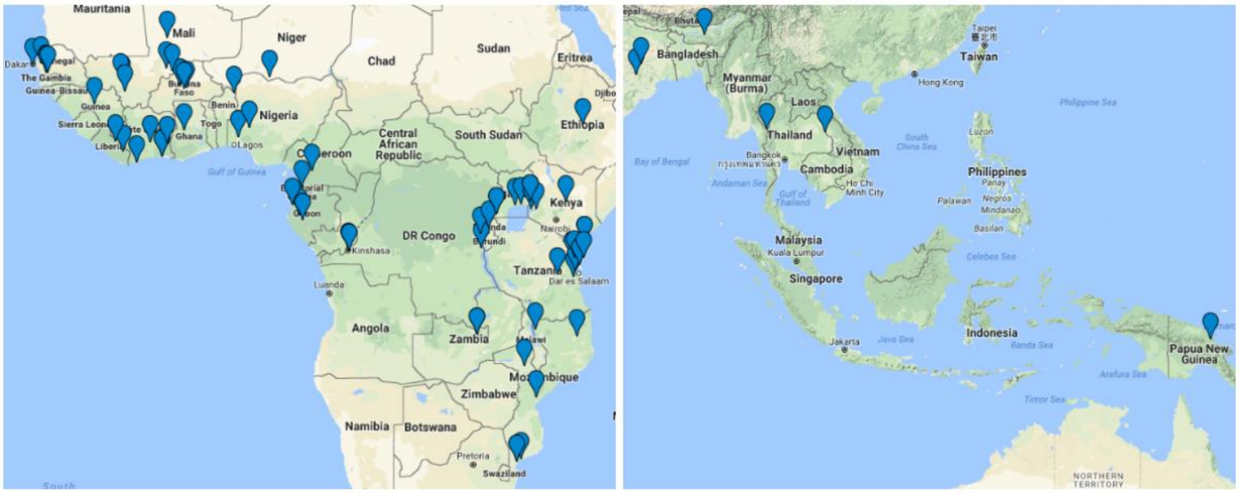

Supplementary Figure 5. Venn diagram of anthropometric measures.

Supplementary Figure 5. Venn diagram of anthropometric measures. % of total population are presented.

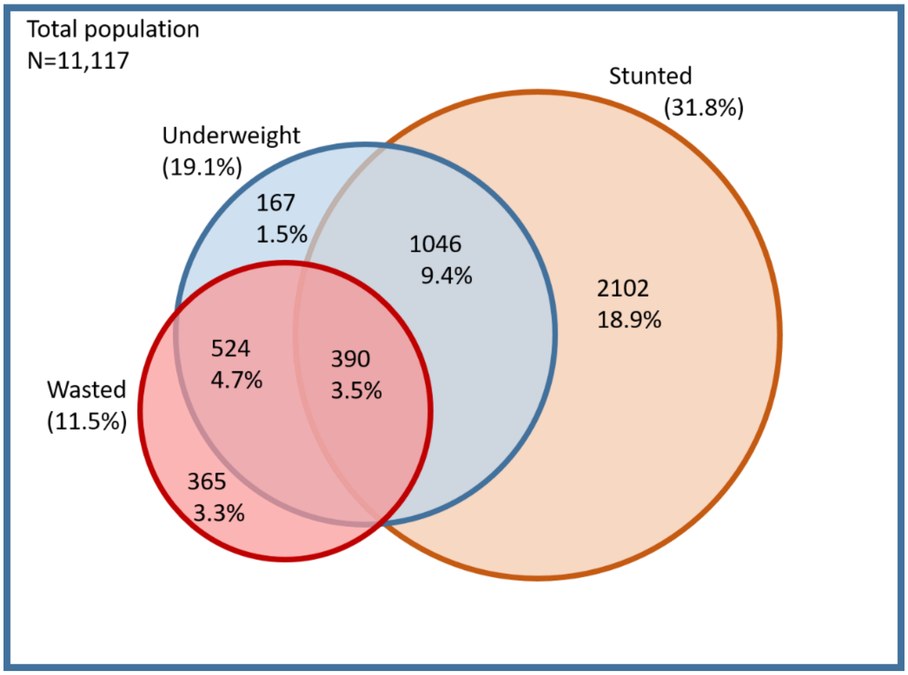

Supplementary Figure 6. Total mg/kg dose by WHZ-score.

Supplementary Figure 6. Total mg/kg dose by WHZ-score. Shown by different ACTs, separately for artemisinin component (left hand side panels) and for partner drugs (right hand side panels).

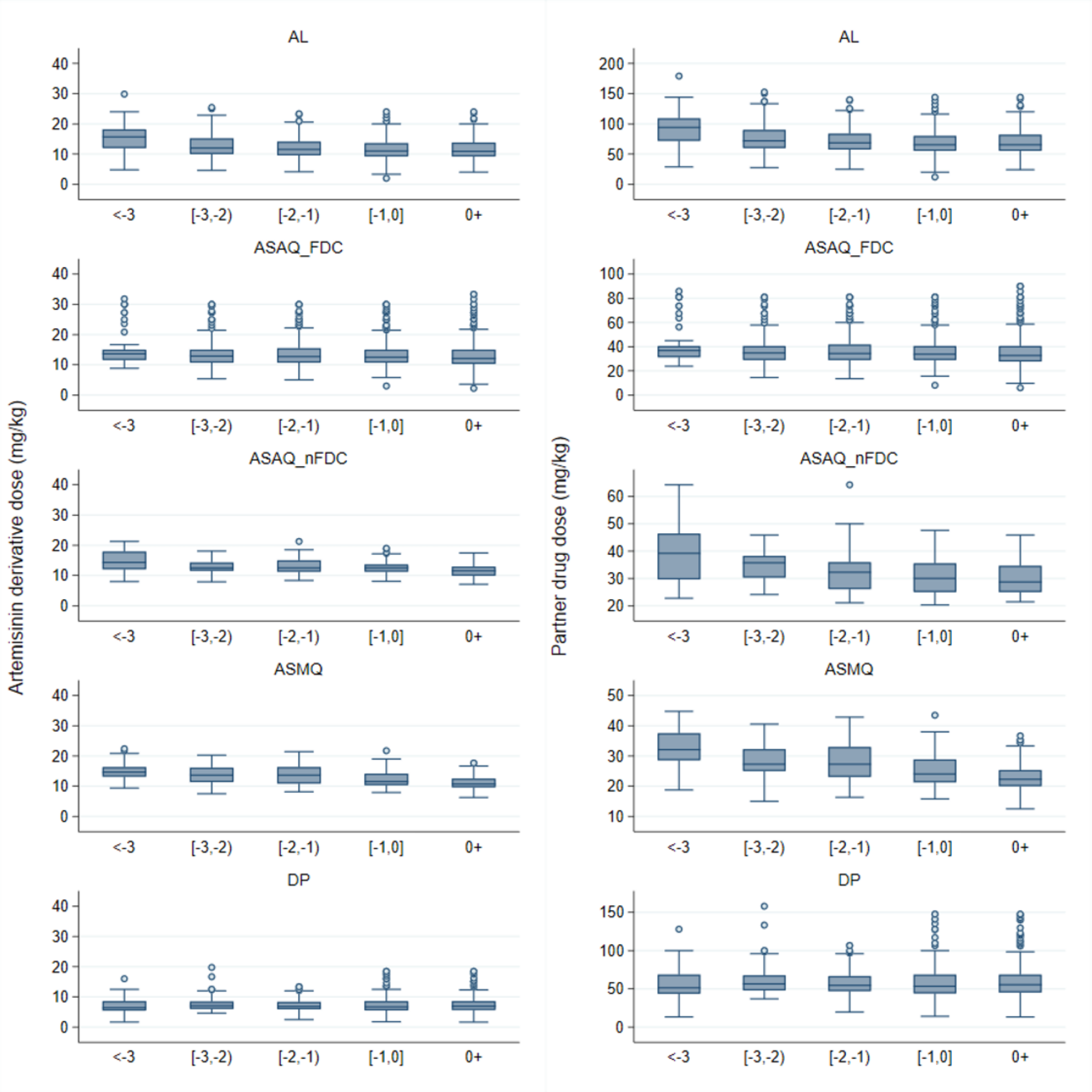

4. Sensitivity analysis

4-1. Assessment of study-specific data and influence of individual cohorts

Supplementary Figure 7. Sensitivity analysis: influence of individual cohorts on estimate of WHZ-score effect

Figures show estimates (and 95% CI) of the effect of weight-for-height z-score when the final multivariable model was refitted after exclusion of data from each study at a time. For comparison, estimates from the full model are also presented in red.

For recrudescence and reinfection, Day-42 models were fitted with interaction terms so separate effect sizes were estimated for intervals: 0-28 days, and 28-42 days (as it was done in the main analysis).

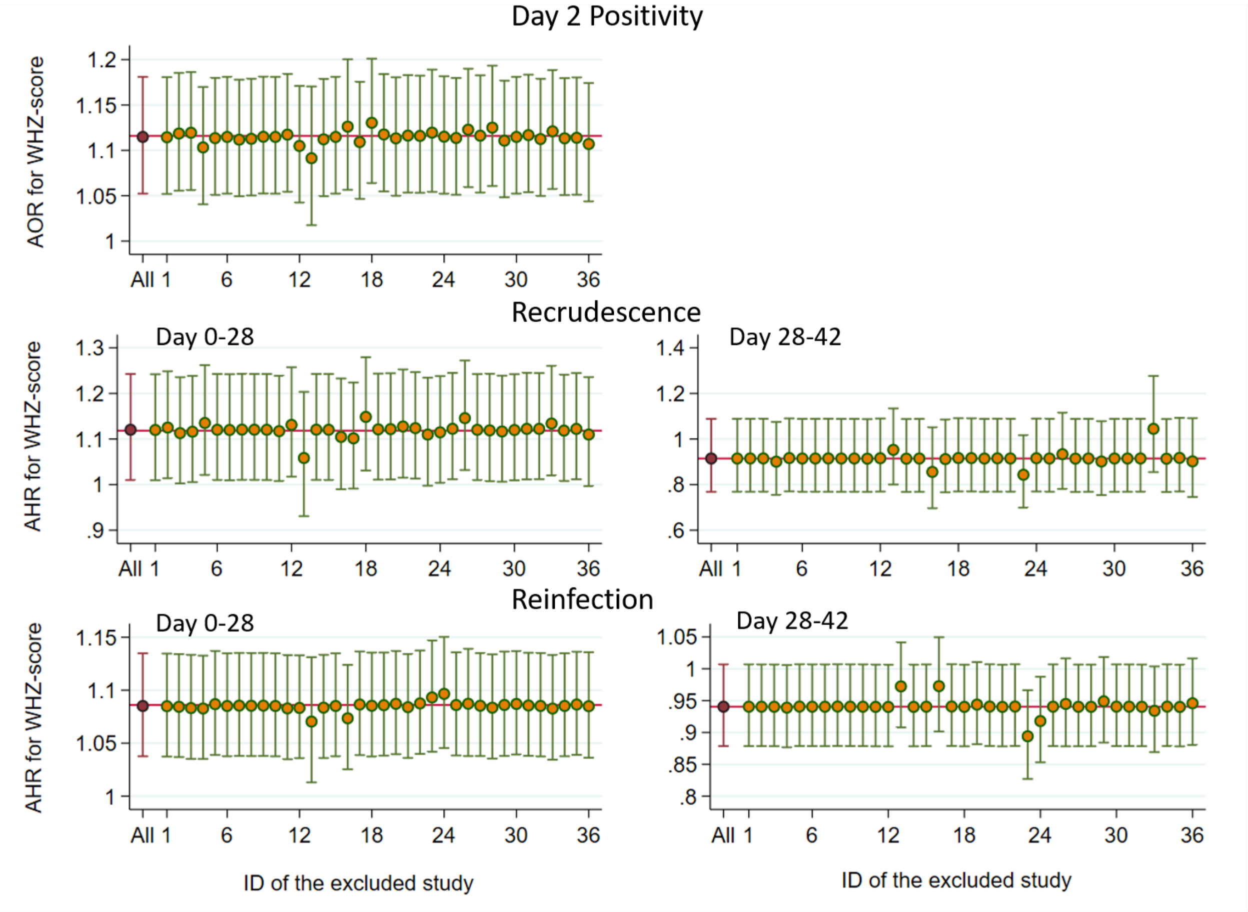

Supplementary Figure 8. Sensitivity analysis: influence of individual cohorts on estimate of HAZ-score effect

Figures show estimates (and 95% CI) of the effect of height-for-age z-score when the final multivariable model was refitted after exclusion of data from each study at a time. For comparison, estimates from the full model are also presented in red.

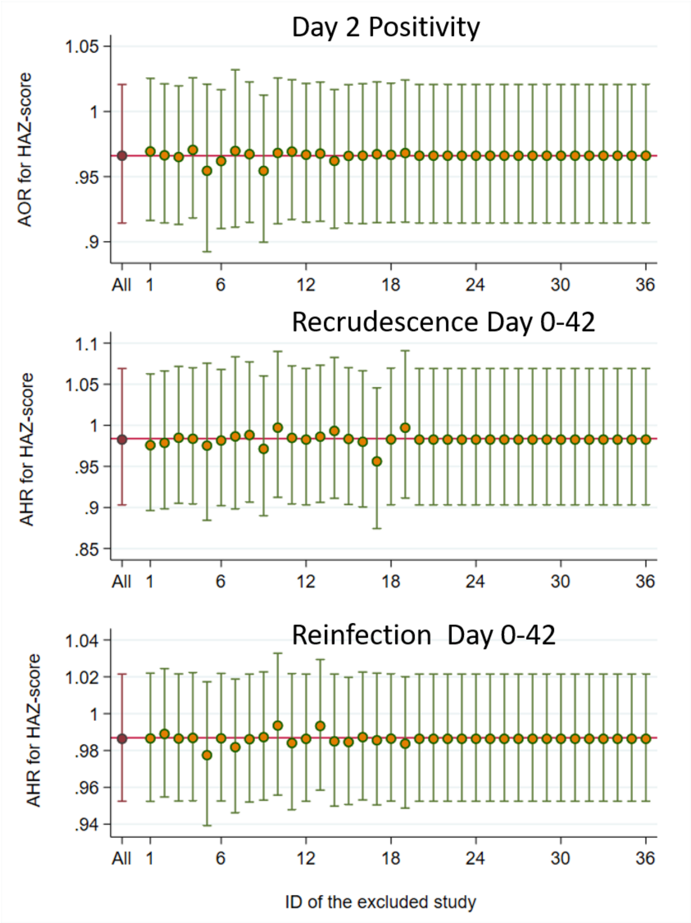

4-2. Subgroup analysis restricted to studies using at least three molecular markers for identification of treatment outcomes

Supplementary Table 7. Independent predictors of treatment outcomes. Results from multivariable Cox regression models with random intercept for study-site, evaluated in 19 studies with 3 molecular markers or microsatellites used.

Parameter		Recrudescence n=9,361			Reinfection n=9,360		
		AHR	95%CI	p-value	AHR	95%CI	p-value
Wasting	WHZ-score[1]	1·14[3]	1·01-1·28	0·033	1·09[4]	1·04-1·14	<0·001
ACT	AL[2]	1			1		
	ASAQ - FDC	0·64	0·44-0·94	0·024	0·86	0·75-0·98	0·026
	ASAQ - nFDC	1·62	0·41-6·42	0·489	1·62	0·48-5·49	0·437
	ASMQ	0·75	0·37-1·50	0·412	0·85	0·67-1·07	0·162
	DP	0·71	0·52-0·96	0·025	0·35	0·30-0·41	<0·001
Age group	<1 year	0·90	0·50-1·63	0·725	0·62	0·51-0·77	<0·001
	1 year	1·41	0·91-2·16	0·121	0·85	0·73-0·99	0·042
	2 years	1·59	1·04-2·43	0·033	0·99	0·85-1·16	0·938
	3 years	1·28	0·81-2·01	0·284	1·00	0·85-1·17	0·985
	4 years	1			1		
Gender	Male				1·09	0·99 -1·20	0·069
	Female				1		
TIA[5]	High	1·17	0·67-2·04	0·575	1·94	1·24-3·03	0·004
	Moderate	0·90	0·55-1·46	0·661	1·07	0·79-1·46	0·659
	Low	1			1		
Log_{10} Parasitaemia (/µL)		1·31	1·03-1·66	0·026			
Anaemia[6]	No	1			1		
	Moderate	1·11	0·83-1·48	0·483	1·07	0·95-1·19	0·256
	Severe	1·65	1·07-2·56	0·024	1·22	1·02-1·47	0·032

[1]estimates shown for 1 unit decrease in weight-for-height z-score; [2]AHR is not constant across the follow-up time - for details see Supplementary table 6; [3]only in first 28 days of follow-up, AHR = 0·91 (0·75-1·10) p=0·320 between 28-42 days; [4]only in first 28 days of follow-up, AHR = 0·95 (0·88-1·02), p=0·131 between 28-42 days; [5]TIA=Transmission Intensity Areas; [6]severe anaemia defined as hb<7g/dL, moderate anaemia as hb between 7 and 10g/dL; [7]fever defined as temperature >37·5ºC or history of fever.

For height-for-age z-score, no association was observed with risk of recrudescence (AHR=0·98, 95%CI 0·82-1·18, p=0·0863) or reinfection (AHR=1·02, 95%CI .96-1·09, p=0·467) in this restricted dataset.

4-3. Multiple imputations of age

Supplementary Table 8. Estimates for the effect of WHZ- and HAZ- scores on treatment outcomes estimated on age imputed dataset. All 36 studies were included for all analyses.

Parameter	N(n) AOR (95% CI) P-value								
	Parasite positivity on D2			Recrudescence[3]			Reinfection[3]		
WASTING									
WHZ (decrease)[1]	10721 (981)	1·12 (1·05-1·18)	<0·001	10758 (305)	1·14 (1·02-1·26)	0·016	10768 (1863)	1·09 (1·04-1·14)	<0·001
Wasted (WHZ<-2)									
No	9562 (890)			9532 (272)			9537 (1586)		
Yes	1159 (91)	1·15 (0·89-1·49)	0·295	1226 (33)	1·15 (0·77-1·74)	0·493	1231 (277)	1·32 (1·14-1·54)	<0·001
WHZ									
≥ 0	4090 (397)			4047 (95)			4049 (577)		
≥ -1 to 0	3367 (316)	1·32 (1·10-1·58)	0·003	3363 (97)	1·27 (0·90-1·79)	0·182	3365 (609)	1·05 (0·91-1·21)	0·507
≥ -2 to -1	2105 (177)	1·33 (1·08-1·65)	0·009	2122 (80)	1·85 (1·29-2·65)	<0·001	2123 (400)	1·15 (0·98-1·34)	0·089
≥ -3 to -2	847 (63)	1·30 (0·95-1·80)	0·105	857 (27)	1·68 (1·02-2·75)	0·041	861 (202)	1·42 (1·17-1·73)	<0·001
< -3	312 (28)	1·51 (0·95-2·40)	0·079	369 (6)	1·08 (0·43-2·74)	0·867	370 (75)	1·33 (1·00-1·79)	0·055
STUNTING									
HAZ (decrease)[2]	10692 (977)	0·98 (0·93-1·03)	0·378	10730 (305)	0·99 (0·91-1·07)	0·758	10741 (1861)	0·98 (0·95-1·02)	0·343
Stunted (HAZ<-2)									
No	7167 (646)			7196 (205)			7202 (1254)		
Yes	3525 (331)	0·98 (0·83-1·16)	0·848	3534 (100)	0·91 (0·71-1·17)	0·472	3539 (607)	0·98 (0·89-1·09)	0·753
HAZ									
≥ 0	1649 (156)			1647 (41)			1648 (278)		
≥ -1 to 0	2420 (212)	0·97 (0·75-1·25)	0·823	2423 (63)	1·02 (0·70-1·49)	0·926	2424 (439)	0·99 (0·85-1·15)	0·900
≥ -2 to -1	3098 (278)	0·94 (0·74-1·20)	0·603	3126 (101)	1·17 (0·82-1·67)	0·378	3130 (537)	0·88 (0·76-1·02)	0·097
≥ -3 to -2	2184 (192)	0·97 (0·74-1·27)	0·819	2212 (62)	0·99 (0·67-1·46)	0·946	2213 (394)	0·93 (0·79-1·09)	0·378
< -3	1341 (139)	0·83 (0·62-1·13)	0·233	1322 (38)	0·95 (0·60-1·50)	0·825	1326 (213)	0·94 (0·78-1·13)	0·493

[1]Estimates shown for 1 unit decrease in weight-for-height z-score (WHZ); [2] Estimates shown for 1 unit decrease in height-for-age z-score (HAZ); [3] For WHZ estimate for 28 days follow-up is shown; N= number of evaluated children; n = number with outcome.

5· Supplementary References

1. WorldWide Antimalarial Resistance Network. Clinical Module: Data Management and Statistical Analysis Plan Version 1.2. 2012 [Available from: http://www.wwarn.org/sites/default/files/ClinicalDMSAP.pdf.
2. Gething PW, Patil AP, Smith DL, Guerra CA, Elyazar IR, Johnston GL, et al. A new world malaria map: Plasmodium falciparum endemicity in 2010. Malaria journal. 2011;10:378.
3. World Health Organization. Malaria surveillance, monitoring & evaluation: a reference manual 2018 [Available from: https://apps.who.int/iris/bitstream/handle/10665/272284/9789241565578-eng.pdf.
4. de Onis M, Garza C, Victora CG, Onyango AW, Frongillo EA, Martines J. The WHO Multicentre Growth Reference Study: planning, study design, and methodology. Food and nutrition bulletin. 2004;25(1 Suppl):S15-26.
5. WHO Multicentre Growth Reference Study Group. WHO Child Growth Standards: Length/height-for-age, weight-for-age, weight-for-length, weight-for-height and body mass index-for-age: Methods and development. Geneva World Health Organization. 2006:312.
6. Lee SJ, Stepniewska K, Anstey N, Ashley E, Barnes K, Binh TQ, et al. The relationship between the haemoglobin concentration and the haematocrit in Plasmodium falciparum malaria. Malaria journal. 2008;7:149.
7. Collett D. Modelling Survival Data in Medical Research. 3rd ed: CRC Press; 2015.
8. Royston P, Altman DG. Regression using fractional polynomials of continuous covariates: parsimonious parametric modelling. Applied Statistics. 1994;43:429-67.
9. Royston P. Multiple imputation of missing values: Further update of ice, with an emphasis on interval censoring. Stata Journal. 2007;7:445-64.
10. Rubin D. Multiple Imputations for Nonresponse in Surveys: NewYork: Wiley; 1987.

6· Members of WWARN ACT Malaria and Malnutrition Study Group

Writing Committee:

Kasia Stepniewska, WorldWide Antimalarial Resistance Network, Oxford, UK and Centre for Tropical Medicine and Global Health, Nuffield Department of Medicine, University of Oxford, UK; **Sheila Isanaka**, Harvard T.H. Chan School of Public Health, Boston, US; **Karen I Barnes**, WorldWide Antimalarial Resistance Network, Oxford, UK and University of Cape Town, Cape Town, South Africa; **Philippe J Guerin**, WorldWide Antimalarial Resistance Network, Oxford, UK and Centre for Tropical Medicine and Global Health, Nuffield Department of Medicine, University of Oxford, UK

Members of WWARN ACT Malaria and Malnutrition Study Group:

Richard Allan, The MENTOR Initiative, Haywards Heath, West Sussex, UK; **Anupkumar R Anvikar**, National Institute of Malaria Research, New Delhi, India; **Thomas A Anyorigiya**, Navrongo Health Research Centre, Navrongo, Ghana and C. K. Tedam University of Technology and Applied Sciences, Navrongo, Ghana; **Elizabeth A**

Ashley, Centre for Tropical Medicine and Global Health, Nuffield Department of Medicine, University of Oxford and Lao-Oxford-Mahosot Hospital-Wellcome Trust Research Unit, Microbiology Laboratory, Mahosot Hospital, Vientiane, Laos; **Quique Bassat**, ISGlobal, Hospital Clinic - Universitat de Barcelona, Barcelona, Spain and Centro de Investigação em Saúde de Manhiça (CISM), Maputo, Mozambique and ICREA, Barcelona, Spain; **Elisabeth Baudin**, Epicentre, Paris; **Anders Bjorkman**, Department of Global Public Health , Karolinska Institute, Stockholm, Sweden; **Maryline Bonnet**, Université de Montpellier/TransVIHMI/IRD/INSERM, Montpellier, France and Epicentre, Mbarara, Uganda; **Caroline Boulton**, Novartis; **Teun Bousema**, Department of Infection and Immunity, London School of Hygiene and Tropical Medicine, London, UK and Department of Medical Microbiology, Radboud University Nijmegen Medical Centre, Njimegen, the Netherlands; **Gwenaelle Carn**, Drugs for Neglected Diseases Initiative, Geneva, Switzerland; **Verena I Carrara**, Shoklo Malaria Research Unit, Mahidol-Oxford Tropical Medicine Research Unit, Faculty of Tropical Medicine, Mahidol University, Mae Sot, Thailand and Mahidol Oxford Research Unit, Faculty of Tropical Medicine, Mahidol University, Bangkok, Thailand and Institute of Global Health, Faculty of Medicine, University of Geneva, Geneva, Switzerland; **Umberto D'Alessandro**, MRC Unit The Gambia at the London School of Hygiene and Tropical Medicine, London, UK; **Timothy ME Davis**, Medical School, University of Western Australia, Fremantle Hospital, Fremantle, Western Australia, Australia; **Lise Denoeud-Ndam**, Epicentre, Paris, France and Research Department, Elizabeth Glaser Pediatric AIDS Foundation, Geneva, Switzerland; **Meghna Desai**, U.S. CDC, Center for Global Health, Division of Global Health Protection, New Delhi, India; **Abdoulaye A Djimde** , Malaria Research and Training Center, Faculty of Pharmacy, University of Science, Techniques and Technologies of Bamako, Bamako, Mali; **Grant Dorsey**, Department of Medicine, University of California San Francisco, San Francisco, CA, USA; **Jean-François Etard**, Epicentre, Paris and TransVIHMI, Institut de Recherche pour le Développement, Université de Montpellier, INSERM, Montpellier, France; **Catherine Falade**, Institute for Advanced Medical Research and Training (IMRAT), College of Medicine, University of Ibadan, Ibadan, Nigeria; **Caterina Fanello**, Centre for Tropical Medicine and Global Health, Nuffield Department of Medicine, University of Oxford and Mahidol Oxford Research Unit, Faculty of Tropical Medicine, Mahidol University, Bangkok, Thailand; **Oumar Gaye**, Department of Medical Parasitology, Medical Faculty, Université Cheikh Anta Diop, Dakar, Senegal; **Raquel Gonzalez**, Centro de Investigação em Saude de Manhiça, Manhiça, Mozambique and Barcelona Institute for Global Health (ISGlobal), Hospital Clínic- Universitat de Barcelona, Spain; **Francesco Grandesso**, Epicentre, Paris, France; **Anastasia D Grivoyannis**, University of Washington, Seattle, WA, USA and Johns Hopkins

University School of Medicicne, Baltimore, MD, USA; **Rebecca F Grais**, Epicentre, Paris, France; **Georgina S Humphreys**, WorldWide Antimalarial Resistance Network, Oxford, UK; **Deus S Ishengoma**, National Institute for Medical Research (NIMR), Dar es Salaam, Tanzania; **Corine Karema**, Quality and Equity Healthcare, Kigali, Rwanda and Swiss Tropical & Public Health Institute, Basel, Switzerland; **Kassoum Kayentao**, Malaria Research and Training Centre, Department of Epidemiology of Parasitologic Diseases, Faculty of Medicine, Pharmacy and Dentistry, University of Bamako, Mali; **Kalynn Kennon**, WorldWide Antimalarial Resistance Network, Oxford, UK and Centre for Tropical Medicine and Global Health, Nuffield Department of Medicine, University of Oxford, UK; **Peter G Kremsner**, Centre de Recherches Médicales de Lambaréné, Lambaréné, Gabon and Institute for Tropical Medicine, Universität Tübingen, Tübingen, Germany; **Moses Laman**, Papua New Guinea Institute of Medical Research, Goroka, Papua New Guinea and School of Medicine and Pharmacology, University of Western Australia, Fremantle Hospital, Fremantle, Western Australia, Australia; **Ibrahim M Laminou**, Centre de Recherche Médicale et Sanitaire, Niamey, Niger; **Eusebio Macete**, Centro de Investigação em Saúde de Manhiça (CISM), Maputo, Mozambique and Instituto Nacional de Saúde (INS), Maputo, Mozambique; **Andreas Martensson**, Department of Women's and Children's Health, Global health & Migration, Uppsala University, Uppsala, Sweden; **Mayfong Mayxay**, Lao-Oxford-Mahosot Hospital, Wellcome Trust Research Unit (LOMWRU), Microbiology Laboratory, Mahosot Hospital, Vientiane, Lao PDR and Institute of Research and Education Development, UHS, Ministry of Health, Vientiane, Lao PDR and Centre for Tropical Medicine and Global Health, Nuffield Department of Clinical Medicine, University of Oxford, Oxford, UK; **Hervé IB Menan**, Department of Parasitology, Faculty of Pharmacy, University of Cocody, Abidjan, Côte d'Ivoire; **Clara Menéndez**, Barcelona Institute for Global Health (ISGlobal) Hospital Clinic, University of Barcelona, Spain and CISM - Manhiça Health Research Center, Manhiça, Mozambique; **Brioni R Moore**, Curtin Medical School, Curtin University, Australia and School of Medicine and Pharmacology, The University of Western Australia, Crawley, Australia and Wesfarmers Centre of Vaccines & Infectious Diseases, Telethon Kids Institute, Australia; **Carolyn Nabasumba**, Epicentre, Paris, France and Faculty of Medicine, Mbarara University of Science and Technology, Mbarara, Uganda; **Jean-Louis Ndiaye**, Parasitology and Mycology Department, Research and Training Unit in Health Sciences, Université Iba Der Thiam, Thies, Senegal; **Abel Nhama**, Centro de Investigação Em Saúde de Manhiça (CISM), Maputo, Mozambique and Instituto Nacional de Saúde (INS), Maputo, Mozambique; **Francois Nosten**, Shoklo Malaria Research Unit, Mahidol-Oxford Tropical Medicine Research Unit, Faculty of Tropical Medicine, Mahidol University, Mae Sot, Thailand and Centre for Tropical

Medicine and Global Health, Nuffield Department of Medicine, University of Oxford, Oxford, UK; **Marie Onyamboko**, Faculty of Tropical Medicine, Mahidol University, Bangkok, Thailand and Kinshasa School of Public Health, University of Kinshasa, Kinshasa, Democratic Republic of the Congo; **Aung Pyae Phyo**, Shoklo Malaria Research Unit, Mahidol-Oxford Tropical Medicine Research Unit, Faculty of Tropical Medicine, Mahidol University, Mae Sot, Thailand and Centre for Tropical Medicine and Global Health, Nuffield Department of Medicine, University of Oxford, United Kingdom; **Michael Ramharter**, Department of Tropical Medicine Bernhard Nocht Institute for Tropical Medicine and I. Dep. of Medicine, University Medical Center Hamburg-Eppendorf; **Philip J Rosenthal**, Department of Medicine, University of California San Francisco, San Francisco, CA, USA; **Birgit Schramm**, Epicentre, Paris, France; **Yagya D Sharma**, Department of Biotechnology, All India Institute of Medical Sciences, New Delhi, India; **Sodiomon B Sirima**, Centre National de Recherche et de Formation sur le Paludisme (CNRFP), Ouagadougou, Burkina Faso; **Nathalie Strub-Wourgaft**, Drugs for Neglected Diseases Initiative, Geneva, Switzerland; **Khadime Sylla**, Service de Parasitologie-Mycologie Médicale, Université Cheikh Anta Diop, Dakar, Senegal; **Ambrose O Talisuna**, University of Oxford/KEMRI/Wellcome Trust Research Programme, Nairobi, Kenya; **Emmanuel A Temu**, The MENTOR Initiative, Haywards Heath, West Sussex, UK and Swiss Tropical and Public Health Institute, Basel, Switzerland and RBM Partnership to end Malaria, Geneva, Switzerland; **Julie I Thwing**, CDC Division of Parasitic Diseases and Malaria, CDC, Atlanta, GA, USA; **Halidou Tint**o, Institut de Recherche en Sciences de la Santé (IRSS), Nanoro, Burkina Faso and Centre Muraz, Bobo-Dioulasso, Burkina Faso; **Giovanni Valentini**, Alfasigma (formerly Sigma-tau); **Nicholas J White**, Mahidol Oxford Reasearch Unit, Bangkok, Thailand and Centre for Tropical Medicine and Global Health, Nuffield Department of Medicine, University of Oxford, Oxford, UK; **Adoke Yeka**, Uganda Malaria Surveillance Project, Kampala, Uganda and Ministry of Health, Kampala, Uganda
